# Genome-wide analysis of the carotenoid cleavage dioxygenases gene family in *Forsythia suspensa*: Expression profile and cold and drought stress responses

**DOI:** 10.3389/fpls.2022.998911

**Published:** 2022-09-20

**Authors:** Xiao-Liang Zhao, Ya-Lin Yang, He-Xiao Xia, Yong Li

**Affiliations:** ^1^School of Basic Medicine, Xinxiang Medical University, Xinxiang, China; ^2^Innovation Platform of Molecular Biology, College of Landscape and Art, Henan Agricultural University, Zhengzhou, China; ^3^State Key Laboratory of Tree Genetics and Breeding, Chinese Academy of Forestry, Beijing, China

**Keywords:** cold stress, drought stress, *CCD* family, gene expression, *Forsythia suspensa*

## Abstract

*Forsythia suspensa* is a famous ornamental and medicinal plant in Oleaceae. *CCD* family is involved in the synthesis of pigments, volatiles, strigolactones, and abscisic acid (ABA) in plants. In this study, the *CCD* family in *F. suspensa* was analyzed at the genome level. A total of 16 members of the *CCD* family were identified, which included 11 members of the carotenoid cleavage dioxygenases (*CCD)* subfamily and 5 members of the 9-cis epoxycarotenoid dioxygenases *(NCED)* subfamily. The expression analysis of different tissues demonstrated that three *FsCCD1* genes might be involved in the synthesis of pigments and volatiles in flowers and fruits. Three *CCD4* genes were effectively expressed in flowers, while only *FsCCD4-3* was effectively expressed in fruits. Comparison of *CCD4* between *Osmanthus fragrans* and *F. suspensa* showed that the structure of FsCCD4-1 is was comparable that of OfCCD4-1 protein, indicating that the protein might be performing, especially in catalyzing the synthesis of β-ionone. However, further comparison of the upstream promoter regions showed that the proteins have major differences in the composition of *cis*-elements, which might be responsible for differences in β-ionone content. On the other hand, four *NCED* genes were significantly up-regulated under cold stress while two were up-regulated in drought stress. The data showed that these genes might be involved in the synthesis of ABA. Taken together, our data improves understanding of the *CCD* family and provides key candidate genes associated with cold and drought stresses in *F. suspensa.*

## Introduction

Ornamental plants play an important role in garden and landscape design and improvement of the environment (Zheng et al., 2021). Due to the wide varieties in ornamental plants, there is no single variety that occupy a large share in the market. Therefore, it is difficult to achieve high economic benefits from planting ornamental plant species. Thus, planting the ornamental plants with both edible and ornamental value, or medicinal and ornamental value, presents an important opportunity for high economic benefit. *Forsythia suspensa* (Thunb.) Vahl. is a famous medicinal and ornamental plant, which belongs to Oleaceae family. *F. suspensa* blooms in early spring, with flowers first and leaves later. *F. suspensa* trees are golden during flowering period, which confers the plants with excellent ornamental effects (Fu et al., 2014). On the other hand, *F. suspensa* fruits contain phillyrin, phillyrin A, α-pinene, β-pinene, terpinen-4-ol, and other volatile components, and it is widely used as a Chinese patent medicine for treatment of colds (Xiang et al., 2021). In fact, recent studies have demonstrated that *F. suspensa* can reduce covid-19 symptoms (Hu et al., 2021). *F. suspensa* is widely cultivated as a medicinal crop in Hebei, Henan, Shanxi, and Shaanxi provinces in China (Li et al., 2022). Because of the important ornamental and medicinal values of *F. suspensa*, its basic and applied research is on the rise (Qiao et al., 2020).

*Carotenoid cleavage dioxygenase* (*CCD*) family is a relatively small gene family in plants, which include *CCD* and 9-cis-epoxy carotenoid dioxygenase (*NCED*) subfamilies (Ohmiya, 2009). This family catalyzes the cleavage of carotenoids with the conjugated double bonds to form various apocarotenoids and their derivatives (Tian et al., 2021). Four members of the *CCD* subfamily were identified in *Arabidopsis*, and included *CCD1*, *CCD4*, *CCD7*, and *CCD8* (Auldridge et al., 2006). Previous data has shown that *CCD1* and *CCD4* are involved in the synthesis of pigments and volatiles (such as α-ionone, β-ionone) in flowers and fruits of many plants (Simkin et al., 2004; Phadungsawat et al., 2020). *CCD7* and *CCD8* are two key genes involved in the synthesis pathway of strigolactones (Umehara et al., 2008), which regulates in the regulation of senescence, root growth, branching and tillering and flower development (Liu et al., 2019). On the other hand, five members of *NCED* subfamily were identified in *Arabidopsis*, which included *NCED2*, *NCED3*, *NCED5*, *NCED6*, and *NCED9* (Auldridge et al., 2006). The *NCED* genes are involved in the synthesis of abscisic acid (ABA) (Frey et al., 2012; Hamzah et al., 2020; Truong et al., 2021). ABA is an important plant hormone that plays major roles in seed development and dormancy, and mediates plant responses to various environmental stresses (Seo and Koshiba, 2002). The *CCD* family has been identified in the genome of many crops, vegetables, and flowers, such as *Brassica napus* (Zhou et al., 2020), *Populus trichocarpa* (Wei et al., 2022), *Gossypium* species (Zhang et al., 2021), Cucurbitaceae species (Cheng et al., 2022), and Rosaceae species (Zhang et al., 2021). However, data on the whole genome characterization and expression analysis of the *CCD* family in *F. suspensa* remains scant.

In this study, we identified the *CCD* family members based on the published *F. suspensa* genome (Li et al., 2022). We then analyzed the expression patterns of the *CCD* genes in fruit, stem, leaf and flower tissues as well as the expression responses to cold and drought stresses. The data showed that unlike *F. suspensa, Osmanthus fragrans*, a plant from the same family, has a strong floral fragrance. We further analyzed differences in the *CCD4* gene, a gene associated with the synthesis of β-ionone, between the *F. suspensa* and *O. fragrans*. Therefore, this study provides in-depth data on the number and classification, gene structure, and expression of the *CCD* gene family at the genome level. Besides, our study provides key candidate genes associated with cold and drought stresses in *F. suspensa*.

## Materials and methods

### Data sources and sequence searches

The genome of *F. suspensa* was obtained from the National Center for Biotechnology Information (NCBI, accession no. JAHHPY000000000; Li et al., 2022). The keywords “CCD” and “NCED” were used to search for the *CCD* genes in the annotation file, and then the candidate genes were blasted in NCBI (Altschul et al., 1990)^1^ to identify the REP65 or PLN02258 domain. The genes with conserved REP65 or PLN02258 domains were considered the true *CCD* genes. Physicochemical properties of the CCD protein in *F. suspensa*, such as molecular weight, isoelectric point, amino acid number, fat index, instability index, and hydrophobicity were predicted using the ExPASy online tool (Artimo et al., 2012).^2^ Subcellular localization of the *CCD* genes was predicted by Plant-mPLoc online software (Chou and Shen, 2010),^3^ while the secondary structure was predicted by the online software SOPMA (Geourjon and Deleage, 1995).^4^

According to the IDs of the identified *CCD* genes and the *F. suspensa* genome sequence, the *CCD* genes were mapped on the chromosomes of *F. suspensa*. The chromosome position of the *CCD* genes was visualized using TBtools software (Chen et al., 2020). The genome and protein sequence data of *O. fragrans* were obtained from NCBI (accession no. PRJNA529305; Yang et al., 2018), while the genome and protein sequence data of *Arabidopsis thaliana* were from the Arabidopsis information resource.^5^

On the other hand, the genome and protein sequence data of *Oryza sativa* were obtained from the Rice Genome Annotation Project (Kawahara et al., 2013).^6^

### Phylogenetic relationship and gene structure

Maximum likelihood (ML) tree (Felsenstein, 1996) was constructed to elucidate the phylogenetic relationship of the *CCD* genes based their amino acid sequences. The ML tree was constructed using MEGA 7.0 (Kumar et al., 2016) with the Jones-Taylor-Thornton model (Jones et al., 1992), pairwise deletion option, and 1,000 bootstrap resampling times. The phylogenetic tree was drawn using FigTree v1.4.4 (Rambaut, 2009), while the introns and exons of all the *F. suspensa CCD* genes were visualized using TBtools (Chen et al., 2020). In addition, the protein domains and conserved motifs of all the *F. suspensa CCD* genes were analyzed by the MEME online tool (Bailey et al., 2009).^7^ The protein domains of the *CCD* gene family of *F. suspensa* were visualized by TBtools (Chen et al., 2020), and the analysis value of conserved motifs was set to 10. The upstream 2,000 bp sequences of all the *CCD* genes in *F. suspensa* were extracted using the TBtools (Chen et al., 2020), and the potential *cis*-acting elements of the *CCD* genes were predicted by PlantCARE online software (Thijs et al., 2002).^8^ The predicted results were visualized by the TBtools (Chen et al., 2020). Amino acid sequences of the *CCD4* genes in *O. fragrans* and *F. suspensa* were compared using DNAMAN 6.0 (Lynnon Crop., Quebec, Canada), while conservative domain analysis of the *CCD4* genes of *O. fragrans* and *F. suspensa* was performed using the online software NCBI Conserved Domain Search (Lu et al., 2020).^9^ The possible *cis*-acting elements of in the upstream 2,000 bp sequences in the *CCD4* genes of *O. fragrans* were predicted using the PlantCARE (see text footnote 8; Thijs et al., 2002).

### Expression profile of the carotenoid cleavage dioxygenases genes in different tissues and under cold and drought stresses

Expression patterns of the *CCD* genes in different tissues of *F. suspensa* was extracted from the RNA-seq data in NCBI. The patterns included data from fruits, stems, leaves (accession no. SRR17386487-SRR17386495), and flowers (accession no. SRX11342985, SRX11342993, and SRX11342994). Fresh fruits, stems, leaves from the *F. suspensa* fruits in the harvest season (July) were sampled from three individuals (Li et al., 2022). Flowers at the budding stage (March) were also sampled from three individuals. All the samples were treated with liquid nitrogen, and then kept in the ultra-low temperature refrigerator at −80°C until extraction of RNA. Leaves are often the most sensitive to drought and cold treatments. Therefore, the gene expression data in leaves was used for analysis in our study. Previous studies (Li et al., 2021a,b) showed that Wuzhishan population has the highest cold and drought tolerance when compared with the other three populations. Thus, the gene expression data of the Wuzhishan population was used as a representative. Expression patterns of the *CCD* genes in *F. suspensa* under drought stress were from Wuzhishan population under 80 and 20% soil water content (accession no. SRX7503009, SRX7503010, SRX7503012-SRX7503015; Li et al., 2021a). Expression patterns of the *CCD* genes in *F. suspensa* under cold stress were from Wuzhishan populations at 25 and 4°C (accession no. SRX7440183-SRX7440188; Li et al., 2021b).

The RNA-seq data from *F. suspensa* were further processed. Low-quality reads with more than 50% of bases possessing a value Q ≤ 10 and more than 10% anonymous nucleotides (N) were eliminated from original sequencing data. Fragments per kilobase of transcript per million fragments mapped (FPKM) was used to profile the gene expression in these samples using StringTie (Pertea et al., 2015). The expression patterns of the *F. suspensa CCD* genes in different tissues and in response to drought and cold stresses were visualized using the R package Heatmap. Significantly expressed *CCD* genes in different tissues of *F. suspensa* were analyzed, and a Log_2_FPKM ≥ 1 was used as the threshold. FC ≥ 2 and FDR ≤ 0.05 were used as thresholds to screen for the *CCD* genes involved in drought and cold stress responses.

### Quantitative real-time transcription PCR validation of carotenoid cleavage dioxygenases genes under cold and drought stresses

To verify the expression patterns of 16 *CCD* genes from transcriptomic data under cold and drought stresses, quantitative real-time transcription PCR (*q*RT-PCR) was performed. The primers (Supplementary Table 1) of 16 *CCD* genes for *q*RT-PCR were designed using primer premier 5.0 (Lalitha, 2000). *q*RT-PCR reaction was performed using the TB Green Premix Ex Taq II (TaKaRa, Beijing, China) on the ABI QuantStudio^®^3 Real-Time System (Applied Biosystems, CA, USA). The amplification procedure was as described in Li et al. (2021b). α elongation factor (Rosati et al., 1999) was used as an internal control, and all these reactions with three repeats. The expression levels of the *CCD* genes were calculated by using the 2^–△△Ct^ method (Livak and Schmittgen, 2001).

## Results

### Gene identification and sequence characteristics of the carotenoid cleavage dioxygenases gene family

**FIGURE 1:**
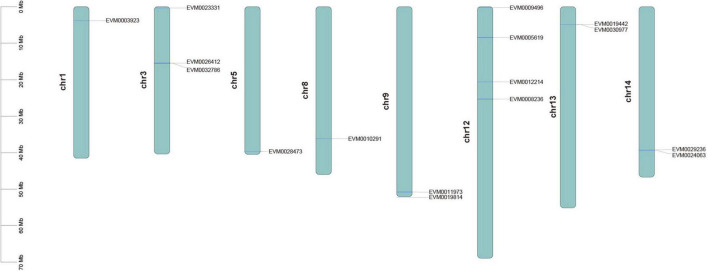
Distribution location of *CCD* gene family in the *Forsythia suspensa* genome.

Our search of the genome annotation files identified a total of 16 possible *CCD* genes from the *F. suspensa* genome. Domain analysis showed that all the genes had the REP65 or PLN02258 conserved domains. Thus, the 16 genes were considered as the real *CCD* genes. CCD proteins in *F. suspensa* demonstrated great variation; where their amino acid length ranged from 123 (FsCCD4-2) to 602 aa (FsCCD7), protein molecular weight ranged from 16.651 (FsCCD4-2) to 67.671 KDa (FsCCD7), while the isoelectric point ranged from 5.11 (FsCCD4-3) to 8.88 (FsCCD4-4) (Supplementary Table 2). Four CCD proteins were localized in the mitochondrion, 4 were in the peroxisome, 2 in the cytoplasm, while the remaining 6 were in the chloroplast (Supplementary Table 2). All the 16 CCD proteins were hydrophilic proteins (Supplementary Table 2). Among the 16 CCD proteins, 5 (FsCCD4-5, FsNCED1-1, FsNCED5-1, FsNCED5-2, and FsNCED6), whose instability index was higher than 40, were predicted to be unstable proteins, while the rest whose value under 40 were stable proteins. The *CCD* genes were randomly distributed on eight chromosomes of *F. suspensa*. Chr12 bore most of the *CCD* genes (25%), while Chr1, Chr5, and Chr8 each accounted for the least (6.25%) of the *CCD* genes (Figure 1).

### Phylogenetic relationship of the carotenoid cleavage dioxygenases proteins in *F. suspensa*

**FIGURE 2:**
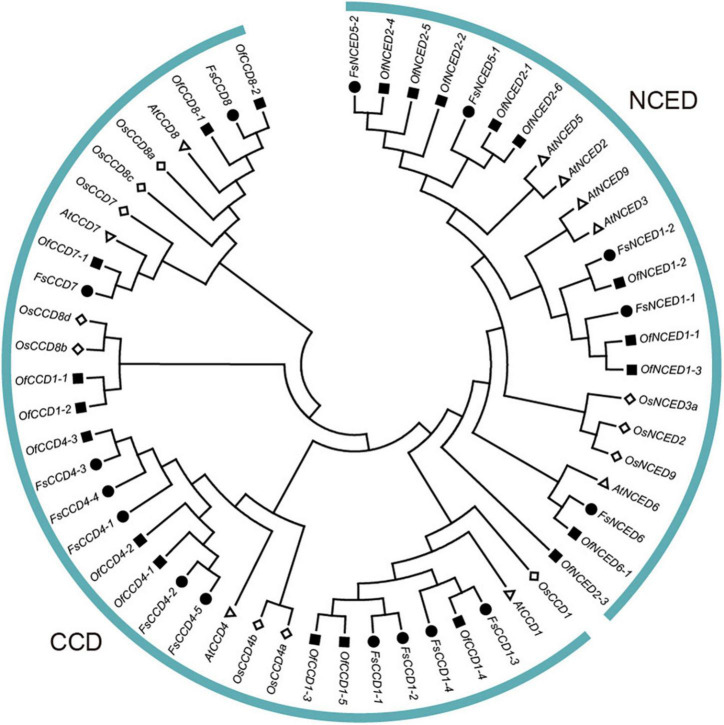
Phylogenetic tree of CCD proteins in *Forsythia suspensa, Osmanthus fragrans*, and *Arabidopsis thaliana*.

To demonstrate the phylogenetic relationship in the CCD proteins of *F. suspensa*, a phylogenetic tree involving 9 CCD proteins in *A. thaliana*, 11 CCD proteins in *O. sativa*, 16 CCD proteins in *F. suspensa*, and 21 CCD proteins in *O. fragrans* was constructed using the ML method. The phylogenetic tree showed that the members of CCD protein family in *F. suspensa* and *O. fragrans* were grouped into two clades, i.e., NCED and CCD clades (Figure 2). NCED proteins were clustered into three subclades, which included FsNCED6, FsNCED1, and FsNCED5. On the other hand, CCD proteins were clustered into three subclades, which included FsCCD1, FsCCD4, and FsCCD7 and FsCCD8. Due to the lack of the NCED5 protein in *O. fragrans*, the two FsNCED5 proteins in *F. suspensa* were clustered together with the four OfNCED2 proteins in *O. fragrans.* Our clustering results showed a close relationship between FsCCD7 and FsCCD8 or FsNCED1 and FsNCED5.

### Gene and protein structure of the carotenoid cleavage dioxygenases gene family

**FIGURE 3:**
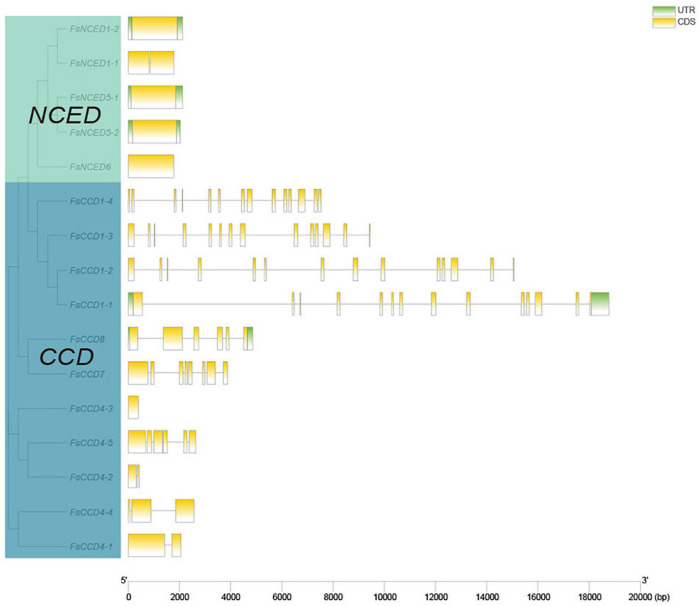
Gene structure analysis of *CCD* genes of *Forsythia suspensa*.

The gene intron and exon structure usually reflect the evolutionary relatedness of the members of a gene family. Here, we analyzed the *CCD* gene sequences of *F. suspensa* and visualized the gene structure using TBtools (Figure 3). The average gene length of the *NCED* subfamily showed minor changes, where four of them had no introns, and only *FsNCED1-1* had a shorter intron. In contrast, there were more changes in the average gene length of the *CCD* subfamily. Four *FsCCD1* genes were significantly longer than the other *CCD* genes. However, they all consisted of 14 exons and 13 introns (Figure 3). Five *FsCCD4* genes were shorter than other *CCD* genes and their number of exons ranged from 1 to 6. *FsCCD7* had 8 exons and 7 introns, while *FsCCD8* had 6 exons and 5 introns.

**FIGURE 4:**
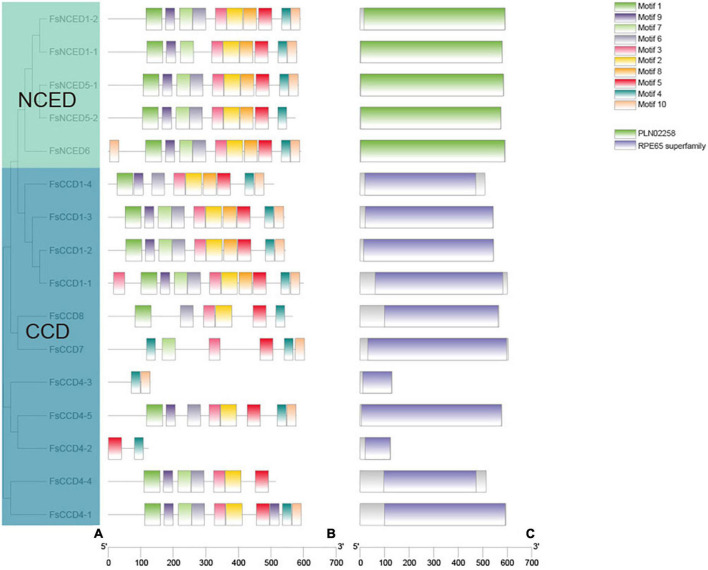
The protein motifs and protein domain analysis of *CCD* genes of *Forsythia suspensa*. **(A)** Phylogenetic tree of *CCD* genes; **(B)** protein motif of *CCD* genes; **(C)** protein domain of *WRKY* genes.

To further analyze the structure and function of the CCD protein in *F. suspensa*, we further identified the conserved domain and motif (Figure 4). Conservative domain analysis showed that the 5 members of NCED subfamily contained the PLN02258 domain, while the CCD subfamily members contained the REP65 domain. Conservative motif analysis showed that the PLN02258 domain was composed of two motif combinations (motif1, motif9, motif7, motif6, motif3, motif2, motif8, motif5, motif4 or motif1, motif9, motif7, motif6, motif3, motif2, motif8, motif5, motif4, motif10). On the other hand, the REP65 domain was composed of multiple combinations. The first combination was motif1, motif9, motif7, motif6, motif3, motif2, motif8, motif5, motif4, motif10, and included FsCCD1-1, FsCCD1-2, FsCCD1-3. FsCCD1-4 lacked motif 7 relative to the first one. FsCCD4-1 contained motif1, motif9, motif7, motif6, motif3, motif2, motif5, motif9, motif4, and motif10. FsCCD4-5 lacked motif 7 and motif 8 relative to the first one. FsCCD4-4 lacked motif 8, motif4, and motif10 relative to the first one. FsCCD7 and FsCCD8 contained six motifs, while FsCCD4-2 and FsCCD4-3 contained two motifs.

Prediction of protein secondary structure of the CCD family showed that the proportion of alpha helices ranged from 7.75% (FsCCD4-3) to 18.15% (FsNCED5-1), while beta turns ranged from 4.33% (FsCCD4-5) to 9.76% (FsCCD4-2). Extended strands ranged from 20.76% (FsCCD7) to 34.88% (FsCCD4-3), while random coils ranged from 47.97% (FsCCD4-2) to 57.50% (FsCCD4-1) (Supplementary Table 3). The results showed that the secondary structure of the CCD protein in *F. suspensa* was mainly composed of extended strands and random coils.

**FIGURE 5:**
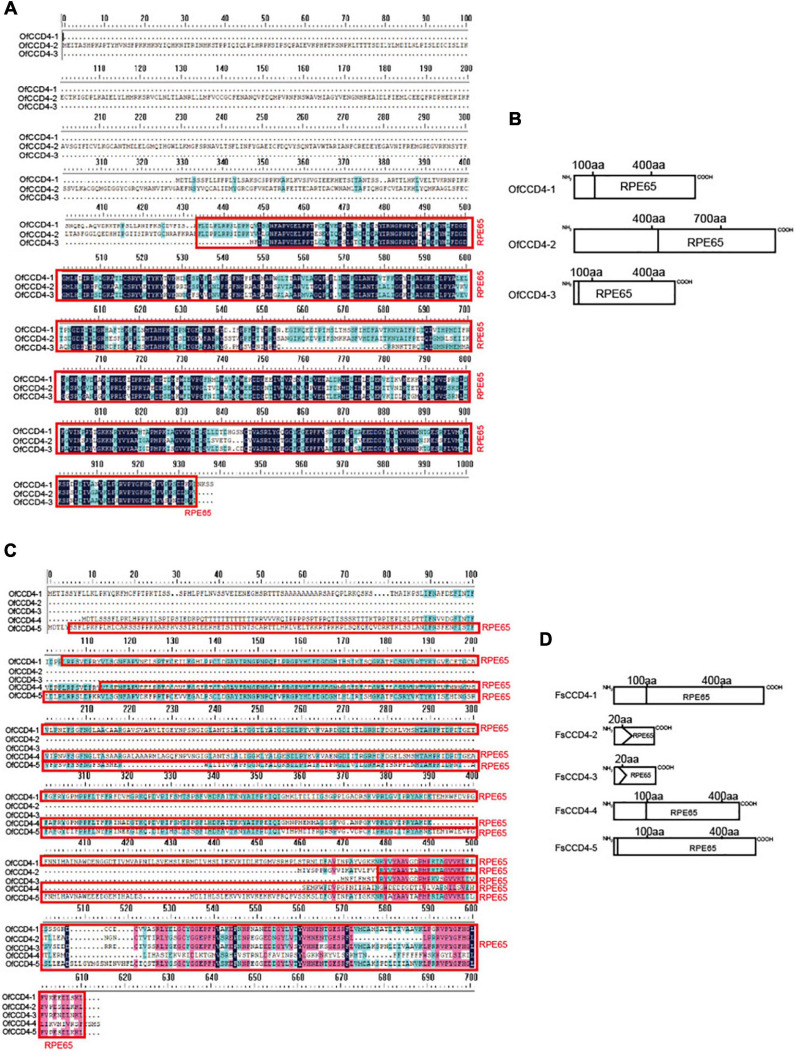
Amino acid sequence and conserved domain of *CCD4* genes of *Forsythia suspensa* and *Osmanthus fragrans*. **(A)** Amino acid sequence of *CCD4* genes in *Osmanthus fragrans*; **(B)** conserved domain of *CCD4* genes in *Osmanthus fragrans*; **(C)** amino acid sequence of *CCD4* genes in *Forsythia suspensa*; **(D)** conserved domain of *CCD4* genes in *Forsythia suspensa.*

A previous study showed that the OfCCD4 protein of *O. fragrans* cleaved β-carotene to produce β-ionone (Zhang et al., 2016). However, *F. suspensa* is not an aromatic plant. Thus, we compared the structural differences in the CCD4 proteins between the *F. suspensa* and *O. fragrans*. Three CCD4 genes were found in *O. fragrans* and five CCD4 genes were in *F. suspensa*. From the protein domain, FsCCD4-1 in *F. suspensa* resembled OfCCD4-1 in *O. fragrans*, while FsCCD4-5 in *F. suspensa* was similar to OfCCD4-3 in *O. fragrans.* However, none of the proteins was similar to the OfCCD4-2 in *O. fragrans* (Figure 5).

### *Cis*-acting elements of the carotenoid cleavage dioxygenases gene family

**FIGURE 6:**
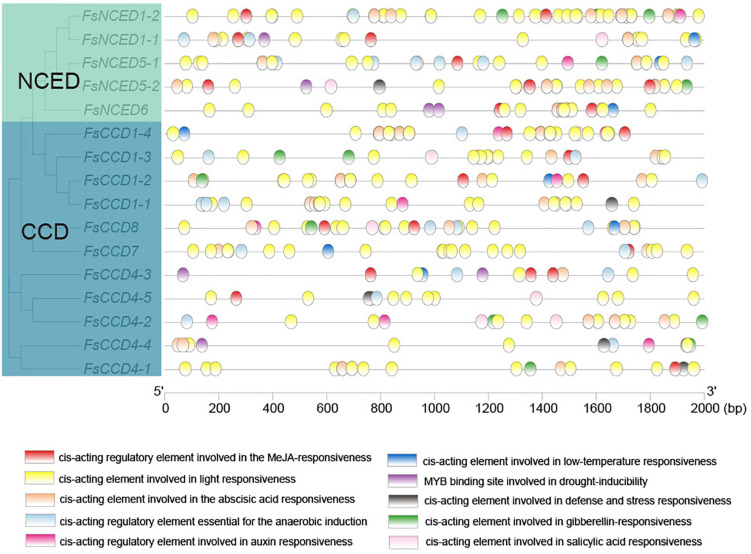
*Cis*-acting elements analysis of the promoters of *CCD* genes of *Forsythia suspensa*.

A total of 417 possible *cis*-acting regulatory elements were identified in the upstream 2,000 bp range of 16 *CCD* genes (Supplementary Table 4 and Figure 6). The results showed that there were many *cis*-acting elements in the promoter region of the *CCD* gene in *F. suspensa*. In addition to many light-responsive elements, the *cis*-acting elements were associated with plant hormones, such as methyl jasmonate (MeJA), ABA, gibberellin (GA), auxin, salicylic acid (SA), and *cis*-acting elements related to stress, such as low temperature, drought, anaerobic environment, and defense and stress, were also found in the *CCD* genes of *F. suspensa*.

**FIGURE 7:**
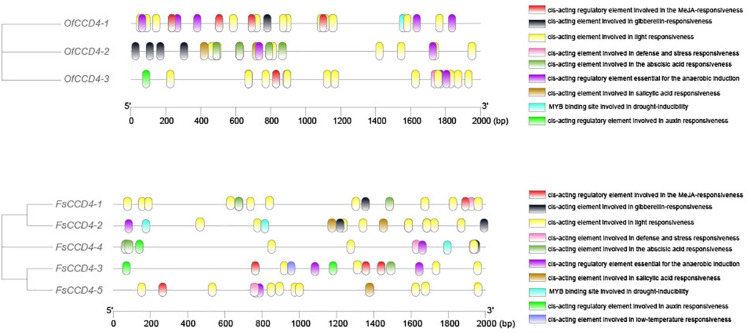
*Cis*-acting elements analysis of the promoters of *CCD4* genes of *Forsythia suspensa* and *Osmanthus fragrans*.

We then compared the *cis*-acting elements upstream of the *CCD4* gene between *O. fragrans* and *F. suspensa*. The data showed that between the similar *FsCCD4-1* and *OfCCD4-1*, *OfCCD4-1* had more anaerobic induction and MeJA-responsiveness *cis*-components compared to *FsCCD4-1*, but had fewer ABA responsiveness *cis*-components than *FsCCD4-1* (Figure 7). Between the similar *FsCCD4-5* and *OfCCD4-3*, *OfCCD4-3* had more auxin responsiveness *cis*-components than *FsCCD4-5*, but had fewer salicylic acid responsiveness *cis*-components than *FsCCD4-5* (Figure 7). The difference in the *cis*-elements upstream of the *CCD4* genes might lead to the difference of β-ionone between *O. fragrans* and *F. suspensa*.

### Expression patterns in the carotenoid cleavage dioxygenases gene in different tissues, cold and drought stresses

**FIGURE 8:**
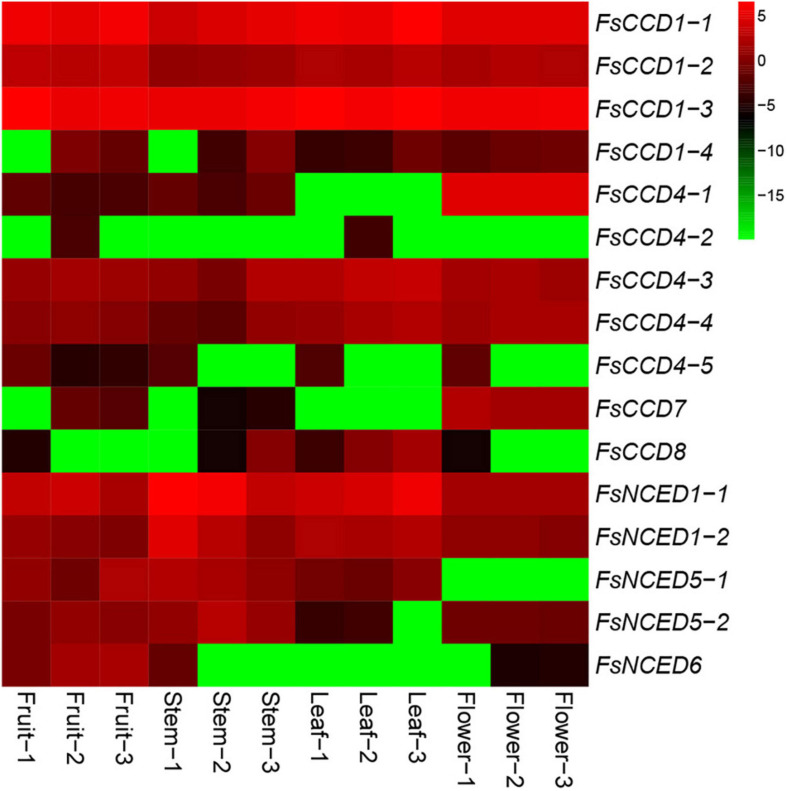
Heat map of *CCD* gene expression (Log_2_FPKM) in different tissues of *Forsythia suspensa*. Fruit-1 to Fruit-3, Stem-1 to Stem-3, Leaf-1 to Leaf-3, and Flower-1 to Flower-3 indicate the three biological replicates from each tissue.

We investigated the expression patterns of the *CCD* genes in fruits, stems, leaves and flowers of *F. suspensa*. The results showed that 7 *CCD* genes were expressed in fruits, 8 in stems, 7 in leaves, and 8 in flowers (Supplementary Table 5 and Figure 8). Our results indicated that about a half of the *CCD* genes might be involved in the development and morphogenesis of fruit, stem, leaf, and flower tissues in *F. suspensa*. In addition, four *FsCCD4* genes were expressed in flowers, and the expression level of *FsCCD4-1* was highest among the four *FsCCD4* genes. Meanwhile, *FsCCD4-1* had specific expression in flowers.

**FIGURE 9:**
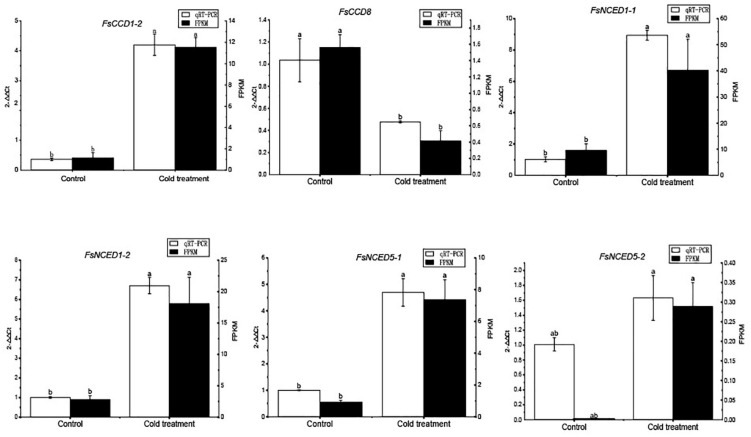
qRT-PCR verification of the *CCD* genes of *Forsythia suspensa* under cold stress. Comparison of *q*RT-PCR (white bar) with RNA-seq data (black bar). The relative changes were calculated with 2^–△△^^Ct^. The relative qRT-PCR expression level is shown on the left *y*-axis. The FPKM from the RNA-Seq data are indicated on the right *y*-axis. The letters above the bars indicates the significance among different samples.

**FIGURE 10:**
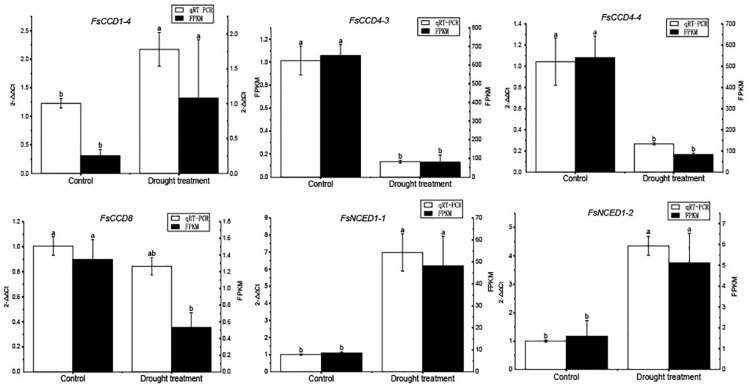
qRT-PCR verification of the *CCD* genes of *Forsythia suspensa* under drought stress. Comparison of *q*RT-PCR (white bar) with RNA-seq data (black bar). The relative changes were calculated with 2^–△△Ct^. The relative qRT-PCR expression level is shown on the left *y*-axis. The FPKM from the RNA-Seq data are indicated on the right *y*-axis. The letters above the bars indicates the significance among different samples.

We further profiled the expression patterns of the *CCD* genes in leaves under cold and drought stresses. Transcriptomic data showed that six *CCD* genes responded to cold stress (Supplementary Table 6 and Supplementary Figure 1), and *q*RT-PCR confirmed these genes responded to cold stress (Figure 9). Of which, 4 (*FsCCD1-2*, *FsCCD8, FsNCED1-1*, and *FsNCED5-1*) had *cis*-elements related to low temperature stress. Six *CCD* genes were significantly differentially expressed under drought stress (Supplementary Table 7 and Supplementary Figure 2), and *q*RT-PCR confirmed all of them responded to drought stress, while *FsCCD8* was only slightly decreased when drought stress (Figure 10). All of them had *cis*-elements with ABA responsiveness (*FsCCD1-4*, *FsCCD4-3*, *FsCCD4-4*, *FsCCD8, FsNCED1-1*, and *FsNCED1-2*), where 3 of the genes had drought-related *cis*-elements involved in *MYB* transcription factors (*FsCCD4-3*, *FsCCD4-4*, and *FsNCED1-1*), and three of them (*FsCCD4-3*, *FsCCD4-4*, and *FsNCED1-1*) had the two kinds of *cis*-elements.

## Discussion

In this study, we systematically analyzed *CCD* genes in *F. suspensa* at the genome level. We profiled the *CCD* genes in *F. suspensa* and *O. fragrans*, and the clustering results supported the division of *CCD* and *NCED* subfamilies of *F. suspensa*. A total of 16 *CCD* genes were identified in *F. suspensa*, which included 11 members in the *CCD* subfamily and 5 members in the *NCED* subfamily., They were more than the number of *CCD* genes (10, 9, 9, 13, 8, 8) identified in six Cucurbitaceae species (Cheng et al., 2022), *Pyrus bretschneideri* (12), *Fragaria vesca* (11), *Prunus mume* (8), and *Prunus persica* (10) (Zhao et al., 2021) and *G. raimondi* (15) (Zhang et al., 2021), but less than the 21 members identified in *O. fragrans*, the 23 members in *Populus trichocarpa* (Wei et al., 2022), 33 and 31 members in *G. hirsutum* and *G. barbadense* (Zhang et al., 2021), 20 members in *Malus domestica* (Zhao et al., 2021), as well as 30 members in *B. napus* (Zhou et al., 2020).

Current studies suggest that the *CCD* family has obvious functional differentiation (Cheng et al., 2022). *CCD1* and *CCD4* are associated with the synthesis of pigments and aromatic substances synthesis in plant flowers and fruits (Xi et al., 2020). The four *FsCCD1* genes were relatively conservative, with little differences in protein length and motif composition. Three *CCD1* genes, *FsCCD1-1*, *FsCCD1-2*, and *FsCCD1-3*, were effectively expressed in fruits and flowers of *F. suspensa*, and might be involved in the synthesis of pigments and volatiles in fruits and flowers. There was also major changes in the *CCD4* gene in *F. suspensa*, with most differences observed in protein length and motif composition. Three *CCD4* genes; *FsCCD4-1*, *FsCCD4-3*, and *FsCCD4-4*, were effectively expressed in *F. suspensa* flowers, while only *FsCCD4-3*, with two motifs, was expressed in fruits.

In *O. fragrans*, *OfCCD4-1*, a famous aromatic plant in the Oleaceae family, which is similar to *F. suspensa* FsCCD4-1 protein domain, has been shown to be involved in the synthesis of β-ionone (Zhang et al., 2016). Therefore, *FsCCD4-1* might be having similar functions, while the other two genes, *FsCCD4-3* and *FsCCD4-4*, might have undergone functional differentiation. Although *FsCCD4-1* was similar to *OfCCD4-1* protein domain, they had great differences in the *cis*-elements in the promoter region. *OfCCD4-1* had *cis*-components with more anaerobic induction and MeJA-responsiveness, but had fewer ABA responsiveness *cis*-components than that of *FsCCD4-1* (Figure 7). In a recent study of *O. fragrans*, the cultivated variety “Zaohuang” (Albus group) had an ethylene response factor binding *cis*-element, which was absent in “Chenghong Dangui” (Aurantiacus group), resulting in a higher content of β-ionone in “Zaohuang” than that in “Chenghong Dangui” (Han et al., 2022). The *cis*-element differences in the upstream promoter region of *FsCCD4-1* and *OfCCD4-1* might have resulted in the difference in the β-ionone content between *F. suspensa* and *O. fragrans.*

*CCD7* and *CCD8* are involved in the synthesis of strigolactones (Umehara et al., 2008), which participate in regulation of aging, root growth, branching, and tillering as well as flower development (Liu et al., 2019). However, our data showed that only *FsCCD7* was effectively expressed in flowers, *CCD7* and *CCD8* were not expressed in stems, leaves and fruits of *F. suspensa*. Here, *FsCCD7* was demonstrated to be involved in the development of *F. suspensa* flowers.

Members of the *NCED* subfamily are involved in the synthesis of ABA (Truong et al., 2021), which is involved in seed development (Seo and Koshiba, 2002). Three *NCED* genes, *FsNCED1-1*, *FsNCED5-1*, and *FsNCED6*, were found to be expressed in *F. suspensa* fruits (Figure 8 and Supplementary Table 5), and were considered to be the candidate genes involved in the development of *F. suspensa* seeds. In addition, ABA was also shown to confer resistance to adverse environment (Seo and Koshiba, 2002). *FsNCED1-1*, *FsNCED*1-2, *FsNCED5-1*, and *FsNCED5-2* in *F. suspensa* were significantly up-regulated under cold stress (Figure 9). Similarly, *FsNCED1-1* and *FsNCED*1-2 genes in *F. suspensa* were significantly up-regulated under drought stress (Figure 10). These genes might be involved in ABA synthesis in *F. suspensa* under cold and drought stress environment, which enhances the ability of *F. suspensa* to withstand cold and drought. The up-regulated expression of the genes in the *NCED* subfamily might be one of the reasons underlying the high cold and drought resistance of *F. suspensa*.

## Conclusion

In this study, a total of 16 members of the *CCD* family were identified, including 11 members of the *CCD* subfamily and 5 members of the *NCED* subfamily. The expression analysis of different tissues showed that three *FsCCD1* genes might be involved into the synthesis of pigments and volatiles in flowers and fruits. Three *CCD4* genes were effectively expressed in flowers, and only one *FsCCD4-3* with two motifs was effectively expressed in fruits. Comparison of the *CCD4* in *Osmanthus fragrans* and *F. suspensa* showed that the structure of FsCCD4-1 was similar to that of OfCCD4-1 protein, indicating that it might have similar functions, especially in catalyzing the synthesis of β-ionone. However, further analysis of the upstream promoter regions showed that they had great differences in the composition of *cis*-elements, which might be associated with differences in the β-ionone content in *F. suspensa* and *O. fragrans*. In addition, four and two *NCED* genes were significantly up-regulated under cold and drought stresses, respectively. These genes might be involved into the synthesis of ABA, and could be used as candidate genes in improving the cold and drought resistance in *F. suspensa*. Taken together, this study improves our understanding of the *CCD* gene family and provides key candidate genes associated with cold and drought stresses in *F. suspensa.*

## Data availability statement

The datasets presented in this study can be found in online repositories. The names of the repository/repositories and accession number(s) can be found in the article/Supplementary material.

## Author contributions

X-LZ and YL coordinated execution of this study. X-LZ performed the RNA-seq analysis. Y-LY performed the gene family analysis. H-XX performed the qPCR experiment. YL wrote the manuscript. All authors have read and agreed to the submission of the manuscript.

## Footnotes

1
https://blast.ncbi.nlm.nih.gov/

2
https://web.expasy.org/cgi-bin/protparam/protparam

3
http://www.csbio.sjtu.edu.cn/bioinf/plant-multi/

4
https://npsa-prabi.ibcp.fr/cgi-bin/secpred_sopma.pl

5
https://www.arabidopsis.org/

6
http://rice.uga.edu/

7
https://meme-suite.org/meme/tools/meme

8
http://bioinformatics.psb.ugent.be/webtools/plantcare/html/

9
https://www.ncbi.nlm.nih.gov/Structure/cdd/wrpsb.cgi
