# Supplementary material for: Genome-wide analysis of the carotenoid cleavage dioxygenases gene family in Forsythia suspensa: Expression profile and cold and drought stress responses
Source: Front Plant Sci. 2022 Sep 20;13:998911. doi: 10.3389/fpls.2022.998911 (PMC9531035; doi:10.3389/fpls.2022.998911)
Supplement: Supplementary file 1 [file Data_Sheet_1.docx]

Supplementary Material

**Genome-wide analysis of the *CCD* gene family in *Forsythia suspensa*: expression profile and cold and drought stress responses**

**Xiao-Liang Zhao^1^, Ya-Lin Yang^2^, He-Xiao Xia^2^,Yong Li^2,3*^**

***Correspondence:** Yong Li: liyongrui1@126.com

# 1 Supplementary Tables

| Table S1 **\| The primers of qRT-PCR for the 16 *CCD* genes.** | | |
| --- | --- | --- |
| Gene | Primers | Sequence |
| *FsCCD1-2* | EVM32786.1-1F | GCAAACTATCGGCTTGCTGG |
| *FsCCD1-2* | EVM32786.1-1R | CCGACTTTCCGGTGTTCTCA |
| *FsCCD1-4* | EVM19814.1-1F | GCAGCCTGGTACCACTTCAA |
| *FsCCD1-4* | EVM19814.1-1R | ACTCTGTCACGAAGAAGGCG |
| *FsCCD4-3* | EVM19442.1-1F | ACGGAGAAGGTTGCTTTGGT |
| *FsCCD4-3* | EVM19442.1-1R | CCTTGGCAGCTTAACAGCAG |
| *FsCCD4-4* | EVM30977.1-IF | TCGATATGGCCCAATGCCTC |
| *FsCCD4-4* | EVM30977.2-IR | CACTTTTCCGGGATTTGCCC |
| *FsCCD8* | EVM08236.1-1F | GTTTCCGGCACCTGTTTGAC |
| *FsCCD8* | EVM08236.1-1R | CGATTCGATTTGCCGGTGTC |
| *FsNCED1-1* | EVM03923.1-1F | GAAAACGGCAGACCCGAAAG |
| *FsNCED1-1* | EVM03923.1-1R | TCTGCCGGACTGATTGTTCC |
| *FsNCED1-2* | EVM09496.1-1F | GTGTATGTGCGAAATGGGGC |
| *FsNCED1-2* | EVM09496.1-1R | TGAAGTAGACCAAGCCTGCG |
| *FsNCED5-1* | EVM28473.1-1F | CCAGCAAGTGGTGTTCAAGC |
| *FsNCED5-1* | EVM28473.1-1R | TTCCGACCAAGAAGGTTCCG |
| *FsNCED5-2* | EVM05619.1-1F | ATCGCCGGACGTTGAGATAC |
| *FsNCED5-2* | EVM05619.1-1R | ACGAAATACGGTTCACCCCC |

| Table S2 **\| Protein characteristics of the CCD family in F. suspensa.** | | | | | | |  |  |
| --- | --- | --- | --- | --- | --- | --- | --- | --- |
| Gene | Gene ID | Protein length (aa) | molecular weight(kD) | Isoelectric point | Instability index | Aliphatic index | Subcellular localization | Hydrophilicity |
| *FsCCD1-1* | EVM0026412.2 | 599 | 67.799 | 6.53 | 28.39 | 82.82 | Mitochondrion | -0.247 |
| *FsCCD1-2* | EVM0032786.1 | 544 | 61.361 | 5.83 | 32.62 | 80.07 | peroxisome | -0.263 |
| *FsCCD1-3* | EVM0011973.1 | 542 | 61.227 | 6.18 | 32.34 | 81.96 | peroxisome | -0.265 |
| *FsCCD1-4* | EVM0019814.1 | 508 | 57.945 | 6.06 | 36.74 | 80.18 | cytoplasm | -0.323 |
| *FsCCD4-1* | EVM0012214.1 | 593 | 64.921 | 6.22 | 39.52 | 82.55 | peroxisome | -0.098 |
| *FsCCD4-2* | EVM0029236.1 | 123 | 13.651 | 5.62 | 30.41 | 88.62 | chloroplast | -0.027 |
| *FsCCD4-3* | EVM0019442.1 | 129 | 14.446 | 5.11 | 34.55 | 79.92 | chloroplast | -0.243 |
| *FsCCD4-4* | EVM0030977.1 | 513 | 56.695 | 8.88 | 35.04 | 82.65 | cytoplasm | -0.103 |
| *FsCCD4-5* | EVM0024063.1 | 577 | 64.616 | 8.53 | 41.08 | 84.80 | chloroplast | -0.198 |
| *FsCCD7* | EVM0010291.1 | 602 | 67.671 | 6.50 | 39.77 | 82.74 | chloroplast | -0.276 |
| *FsCCD8* | EVM0008236.1 | 565 | 62.951 | 6.92 | 35.01 | 78.85 | peroxisome | -0.334 |
| *FsNCED1-1* | EVM0003923.1 | 579 | 64.504 | 6.48 | 42.38 | 72.90 | Mitochondrion | -0.377 |
| *FsNCED1-2* | EVM0009496.1 | 590 | 66.106 | 6.04 | 37.87 | 83.29 | Mitochondrion | -0.286 |
| *FsNCED5-1* | EVM0028473.1 | 584 | 65.079 | 6.13 | 44.15 | 82.64 | chloroplast | -0.353 |
| *FsNCED5-2* | EVM0005619.1 | 574 | 64.081 | 7.69 | 41.17 | 82.40 | Mitochondrion | -0.316 |
| *FsNCED6* | EVM0023331.1 | 591 | 65.356 | 8.78 | 40.08 | 89.85 | chloroplast | -0.169 |

| Table S3 **\| The secondary structure of CCD protein.** | | | | | |
| --- | --- | --- | --- | --- | --- |
| Gene | Gene ID | Ratio of alpha helix | Ratio of beta turn | Ratio of extended strand | Ratio of random coil |
| FsCCD1-1 | EVM0026412.2 | 16.86 | 5.51 | 26.71 | 50.92 |
| FsCCD1-2 | EVM0032786.1 | 17.46 | 6.07 | 24.45 | 52.02 |
| FsCCD1-3 | EVM0011973.1 | 15.50 | 6.09 | 24.91 | 53.51 |
| FsCCD1-4 | EVM0019814.1 | 14.17 | 6.10 | 24.61 | 55.12 |
| FsCCD4-1 | EVM0012214.1 | 15.35 | 5.90 | 21.25 | 57.50 |
| FsCCD4-2 | EVM0029236.1 | 8.13 | 9.76 | 34.15 | 47.97 |
| FsCCD4-3 | EVM0019442.1 | 7.75 | 6.98 | 34.88 | 50.39 |
| FsCCD4-4 | EVM0030977.1 | 15.59 | 6.82 | 23.20 | 54.39 |
| FsCCD4-5 | EVM0024063.1 | 17.85 | 4.33 | 22.53 | 55.29 |
| FsCCD7 | EVM0010291.1 | 18.11 | 5.48 | 20.76 | 55.65 |
| FsCCD8 | EVM0008236.1 | 14.87 | 5.84 | 22.83 | 56.46 |
| FsNCED1-1 | EVM0003923.1 | 16.58 | 5.18 | 23.66 | 54.58 |
| FsNCED1-2 | EVM0009496.1 | 17.29 | 6.27 | 23.73 | 52.71 |
| FsNCED5-1 | EVM0028473.1 | 18.15 | 4.62 | 22.95 | 54.28 |
| FsNCED5-2 | EVM0005619.1 | 16.90 | 5.57 | 24.04 | 53.48 |
| FsNCED6 | EVM0023331.1 | 14.72 | 6.43 | 22.67 | 56.18 |
|  |  |  |  |  |  |

| **Table S4** **\| Cis-acting element of CCD genes in *F. suspensa.*** | | | | | |
| --- | --- | --- | --- | --- | --- |
| Gene ID | Start location | End location | motif name | motif_sequence | Cis-acting element |
| EVM0003923.1 | 1713 | 1759 | G-Box | CACGTG | cis-acting regulatory element involved in light responsiveness |
| EVM0003923.1 | 175 | 221 | G-box | TACGTG | cis-acting regulatory element involved in light responsiveness |
| EVM0003923.1 | 267 | 313 | G-box | TACGTG | cis-acting regulatory element involved in light responsiveness |
| EVM0003923.1 | 1713 | 1759 | G-box | CACGTG | cis-acting regulatory element involved in light responsiveness |
| EVM0003923.1 | 1727 | 1776 | G-box | TAACACGTAG | cis-acting regulatory element involved in light responsiveness |
| EVM0003923.1 | 635 | 683 | AE-box | AGAAACTT | part of a module for light response |
| EVM0003923.1 | 163 | 213 | GATA-motif | AAGATAAGATT | part of a light responsive element |
| EVM0003923.1 | 461 | 511 | GATA-motif | AAGATAAGATT | part of a light responsive element |
| EVM0003923.1 | 51 | 97 | ARE | AAACCA | cis-acting regulatory element essential for the anaerobic induction |
| EVM0003923.1 | 293 | 339 | ARE | AAACCA | cis-acting regulatory element essential for the anaerobic induction |
| EVM0003923.1 | 1911 | 1960 | I-box | gGATAAGGTG | part of a light responsive element |
| EVM0003923.1 | 645 | 691 | TCT-motif | TCTTAC | part of a light responsive element |
| EVM0003923.1 | 1948 | 1994 | TCT-motif | TCTTAC | part of a light responsive element |
| EVM0003923.1 | 1306 | 1353 | GT1-motif | GGTTAAT | light responsive element |
| EVM0003923.1 | 176 | 221 | ABRE | ACGTG | cis-acting element involved in the abscisic acid responsiveness |
| EVM0003923.1 | 268 | 313 | ABRE | ACGTG | cis-acting element involved in the abscisic acid responsiveness |
| EVM0003923.1 | 1713 | 1759 | ABRE | CACGTG | cis-acting element involved in the abscisic acid responsiveness |
| EVM0003923.1 | 1714 | 1759 | ABRE | ACGTG | cis-acting element involved in the abscisic acid responsiveness |
| EVM0003923.1 | 252 | 297 | CGTCA-motif | CGTCA | cis-acting regulatory element involved in the MeJA-responsiveness |
| EVM0003923.1 | 744 | 789 | CGTCA-motif | CGTCA | cis-acting regulatory element involved in the MeJA-responsiveness |
| EVM0003923.1 | 252 | 297 | TGACG-motif | TGACG | cis-acting regulatory element involved in the MeJA-responsiveness |
| EVM0003923.1 | 744 | 789 | TGACG-motif | TGACG | cis-acting regulatory element involved in the MeJA-responsiveness |
| EVM0003923.1 | 1599 | 1648 | TCA-element | TCAGAAGAGG | cis-acting element involved in salicylic acid responsiveness |
| EVM0003923.1 | 1942 | 1988 | LTR | CCGAAA | cis-acting element involved in low-temperature responsiveness |
| EVM0003923.1 | 351 | 397 | MBS | CAACTG | MYB binding site involved in drought-inducibility |
| EVM0005619.1 | 1412 | 1458 | G-Box | CACGTG | cis-acting regulatory element involved in light responsiveness |
| EVM0005619.1 | 1569 | 1615 | G-Box | CACGTT | cis-acting regulatory element involved in light responsiveness |
| EVM0005619.1 | 1636 | 1682 | G-Box | CACGTT | cis-acting regulatory element involved in light responsiveness |
| EVM0005619.1 | 1812 | 1858 | G-Box | CACGTG | cis-acting regulatory element involved in light responsiveness |
| EVM0005619.1 | 1282 | 1329 | MRE | AACCTAA | MYB binding site involved in light responsiveness |
| EVM0005619.1 | 43 | 89 | G-box | CACGTC | cis-acting regulatory element involved in light responsiveness |
| EVM0005619.1 | 221 | 267 | G-box | CACGAC | cis-acting regulatory element involved in light responsiveness |
| EVM0005619.1 | 1411 | 1459 | G-box | ACACGTGT | cis-acting regulatory element involved in light responsiveness |
| EVM0005619.1 | 1412 | 1458 | G-box | CACGTG | cis-acting regulatory element involved in light responsiveness |
| EVM0005619.1 | 1812 | 1858 | G-box | CACGTG | cis-acting regulatory element involved in light responsiveness |
| EVM0005619.1 | 995 | 1041 | TCT-motif | TCTTAC | part of a light responsive element |
| EVM0005619.1 | 1913 | 1960 | GARE-motif | TCTGTTG | gibberellin-responsive element |
| EVM0005619.1 | 1280 | 1326 | GT1-motif | GGTTAA | light responsive element |
| EVM0005619.1 | 1521 | 1567 | GT1-motif | GGTTAA | light responsive element |
| EVM0005619.1 | 141 | 186 | CGTCA-motif | CGTCA | cis-acting regulatory element involved in the MeJA-responsiveness |
| EVM0005619.1 | 1333 | 1378 | CGTCA-motif | CGTCA | cis-acting regulatory element involved in the MeJA-responsiveness |
| EVM0005619.1 | 1778 | 1823 | CGTCA-motif | CGTCA | cis-acting regulatory element involved in the MeJA-responsiveness |
| EVM0005619.1 | 43 | 88 | ABRE | ACGTG | cis-acting element involved in the abscisic acid responsiveness |
| EVM0005619.1 | 1412 | 1458 | ABRE | CACGTG | cis-acting element involved in the abscisic acid responsiveness |
| EVM0005619.1 | 1413 | 1458 | ABRE | ACGTG | cis-acting element involved in the abscisic acid responsiveness |
| EVM0005619.1 | 1569 | 1614 | ABRE | ACGTG | cis-acting element involved in the abscisic acid responsiveness |
| EVM0005619.1 | 1636 | 1681 | ABRE | ACGTG | cis-acting element involved in the abscisic acid responsiveness |
| EVM0005619.1 | 1812 | 1858 | ABRE | CACGTG | cis-acting element involved in the abscisic acid responsiveness |
| EVM0005619.1 | 1813 | 1858 | ABRE | ACGTG | cis-acting element involved in the abscisic acid responsiveness |
| EVM0005619.1 | 1330 | 1378 | TGA-box | TGACGTAA | part of an auxin-responsive element |
| EVM0005619.1 | 141 | 186 | TGACG-motif | TGACG | cis-acting regulatory element involved in the MeJA-responsiveness |
| EVM0005619.1 | 1333 | 1378 | TGACG-motif | TGACG | cis-acting regulatory element involved in the MeJA-responsiveness |
| EVM0005619.1 | 1778 | 1823 | TGACG-motif | TGACG | cis-acting regulatory element involved in the MeJA-responsiveness |
| EVM0005619.1 | 774 | 823 | TC-rich repeats | ATTCTCTAAC | cis-acting element involved in defense and stress responsiveness |
| EVM0005619.1 | 594 | 643 | TCA-element | CCATCTTTTT | cis-acting element involved in salicylic acid responsiveness |
| EVM0005619.1 | 1877 | 1924 | TCCC-motif | TCTCCCT | part of a light responsive element |
| EVM0005619.1 | 507 | 553 | MBS | CAACTG | MYB binding site involved in drought-inducibility |
| EVM0008236.1 | 1699 | 1748 | G-box | TAACACGTAG | cis-acting regulatory element involved in light responsiveness |
| EVM0008236.1 | 1700 | 1746 | G-box | CACGTG | cis-acting regulatory element involved in light responsiveness |
| EVM0008236.1 | 315 | 362 | AuxRR-core | GGTCCAT | cis-acting regulatory element involved in auxin responsiveness |
| EVM0008236.1 | 1050 | 1096 | G-Box | CACGTT | cis-acting regulatory element involved in light responsiveness |
| EVM0008236.1 | 1700 | 1746 | G-Box | CACGTG | cis-acting regulatory element involved in light responsiveness |
| EVM0008236.1 | 52 | 98 | Box 4 | ATTAAT | part of a conserved DNA module involved in light responsiveness |
| EVM0008236.1 | 385 | 431 | Box 4 | ATTAAT | part of a conserved DNA module involved in light responsiveness |
| EVM0008236.1 | 508 | 554 | Box 4 | ATTAAT | part of a conserved DNA module involved in light responsiveness |
| EVM0008236.1 | 615 | 661 | Box 4 | ATTAAT | part of a conserved DNA module involved in light responsiveness |
| EVM0008236.1 | 793 | 839 | Box 4 | ATTAAT | part of a conserved DNA module involved in light responsiveness |
| EVM0008236.1 | 797 | 843 | Box 4 | ATTAAT | part of a conserved DNA module involved in light responsiveness |
| EVM0008236.1 | 868 | 914 | Box 4 | ATTAAT | part of a conserved DNA module involved in light responsiveness |
| EVM0008236.1 | 1119 | 1165 | Box 4 | ATTAAT | part of a conserved DNA module involved in light responsiveness |
| EVM0008236.1 | 1202 | 1248 | Box 4 | ATTAAT | part of a conserved DNA module involved in light responsiveness |
| EVM0008236.1 | 1641 | 1687 | Box 4 | ATTAAT | part of a conserved DNA module involved in light responsiveness |
| EVM0008236.1 | 522 | 569 | P-box | CCTTTTG | gibberellin-responsive element |
| EVM0008236.1 | 963 | 1009 | ARE | AAACCA | cis-acting regulatory element essential for the anaerobic induction |
| EVM0008236.1 | 1062 | 1108 | ARE | AAACCA | cis-acting regulatory element essential for the anaerobic induction |
| EVM0008236.1 | 1549 | 1595 | ARE | AAACCA | cis-acting regulatory element essential for the anaerobic induction |
| EVM0008236.1 | 638 | 687 | ACE | GACACGTATG | cis-acting element involved in light responsiveness |
| EVM0008236.1 | 747 | 796 | TCA-element | TCAGAAGAGG | cis-acting element involved in salicylic acid responsiveness |
| EVM0008236.1 | 572 | 617 | TGACG-motif | TGACG | cis-acting regulatory element involved in the MeJA-responsiveness |
| EVM0008236.1 | 904 | 949 | TGACG-motif | TGACG | cis-acting regulatory element involved in the MeJA-responsiveness |
| EVM0008236.1 | 572 | 617 | CGTCA-motif | CGTCA | cis-acting regulatory element involved in the MeJA-responsiveness |
| EVM0008236.1 | 904 | 949 | CGTCA-motif | CGTCA | cis-acting regulatory element involved in the MeJA-responsiveness |
| EVM0008236.1 | 318 | 365 | ABRE | TACGGTC | cis-acting element involved in the abscisic acid responsiveness |
| EVM0008236.1 | 1051 | 1096 | ABRE | ACGTG | cis-acting element involved in the abscisic acid responsiveness |
| EVM0008236.1 | 1700 | 1746 | ABRE | CACGTG | cis-acting element involved in the abscisic acid responsiveness |
| EVM0008236.1 | 1701 | 1746 | ABRE | ACGTG | cis-acting element involved in the abscisic acid responsiveness |
| EVM0008236.1 | 1646 | 1692 | LTR | CCGAAA | cis-acting element involved in low-temperature responsiveness |
| EVM0009496.1 | 1687 | 1736 | Box II | CCACGTGGC | part of a light responsive element |
| EVM0009496.1 | 1688 | 1737 | Box II | CCACGTGGC | part of a light responsive element |
| EVM0009496.1 | 82 | 129 | TCCC-motif | TCTCCCT | part of a light responsive element |
| EVM0009496.1 | 1351 | 1404 | AT1-motif | AATTATTTTTTATT | part of a light responsive module |
| EVM0009496.1 | 282 | 327 | CGTCA-motif | CGTCA | cis-acting regulatory element involved in the MeJA-responsiveness |
| EVM0009496.1 | 1393 | 1438 | CGTCA-motif | CGTCA | cis-acting regulatory element involved in the MeJA-responsiveness |
| EVM0009496.1 | 378 | 424 | GT1-motif | GGTTAA | light responsive element |
| EVM0009496.1 | 965 | 1012 | GT1-motif | GGTTAAT | light responsive element |
| EVM0009496.1 | 966 | 1012 | GT1-motif | GGTTAA | light responsive element |
| EVM0009496.1 | 1439 | 1485 | GT1-motif | GGTTAA | light responsive element |
| EVM0009496.1 | 1292 | 1338 | Box 4 | ATTAAT | part of a conserved DNA module involved in light responsiveness |
| EVM0009496.1 | 1471 | 1517 | Box 4 | ATTAAT | part of a conserved DNA module involved in light responsiveness |
| EVM0009496.1 | 1509 | 1555 | Box 4 | ATTAAT | part of a conserved DNA module involved in light responsiveness |
| EVM0009496.1 | 1230 | 1277 | P-box | CCTTTTG | gibberellin-responsive element |
| EVM0009496.1 | 1775 | 1822 | P-box | CCTTTTG | gibberellin-responsive element |
| EVM0009496.1 | 1502 | 1551 | ACE | CTAACGTATT | cis-acting element involved in light responsiveness |
| EVM0009496.1 | 1580 | 1627 | GARE-motif | TCTGTTG | gibberellin-responsive element |
| EVM0009496.1 | 657 | 703 | G-box | CACGAC | cis-acting regulatory element involved in light responsiveness |
| EVM0009496.1 | 770 | 819 | G-box | GCCACGTGGA | cis-acting regulatory element involved in light responsiveness |
| EVM0009496.1 | 772 | 818 | G-box | CACGTG | cis-acting regulatory element involved in light responsiveness |
| EVM0009496.1 | 830 | 880.5 | G-box | ACACGTG(G/t)CACC | cis-acting regulatory element involved in light responsiveness |
| EVM0009496.1 | 835 | 881 | G-box | CACGTC | cis-acting regulatory element involved in light responsiveness |
| EVM0009496.1 | 1576 | 1622 | G-box | CACGTC | cis-acting regulatory element involved in light responsiveness |
| EVM0009496.1 | 1687 | 1736 | G-box | GCCACGTGGA | cis-acting regulatory element involved in light responsiveness |
| EVM0009496.1 | 1689 | 1735 | G-box | CACGTG | cis-acting regulatory element involved in light responsiveness |
| EVM0009496.1 | 1861 | 1909 | G-box | ACACGTGT | cis-acting regulatory element involved in light responsiveness |
| EVM0009496.1 | 1862 | 1908 | G-box | CACGTG | cis-acting regulatory element involved in light responsiveness |
| EVM0009496.1 | 469 | 517 | AE-box | AGAAACAA | part of a module for light response |
| EVM0009496.1 | 772 | 818 | G-Box | CACGTG | cis-acting regulatory element involved in light responsiveness |
| EVM0009496.1 | 1689 | 1735 | G-Box | CACGTG | cis-acting regulatory element involved in light responsiveness |
| EVM0009496.1 | 1862 | 1908 | G-Box | CACGTG | cis-acting regulatory element involved in light responsiveness |
| EVM0009496.1 | 772 | 818 | ABRE | CACGTG | cis-acting element involved in the abscisic acid responsiveness |
| EVM0009496.1 | 773 | 818 | ABRE | ACGTG | cis-acting element involved in the abscisic acid responsiveness |
| EVM0009496.1 | 835 | 880 | ABRE | ACGTG | cis-acting element involved in the abscisic acid responsiveness |
| EVM0009496.1 | 1576 | 1621 | ABRE | ACGTG | cis-acting element involved in the abscisic acid responsiveness |
| EVM0009496.1 | 1687 | 1736 | ABRE | GCCGCGTGGC | cis-acting element involved in the abscisic acid responsiveness |
| EVM0009496.1 | 1689 | 1735 | ABRE | CACGTG | cis-acting element involved in the abscisic acid responsiveness |
| EVM0009496.1 | 1690 | 1735 | ABRE | ACGTG | cis-acting element involved in the abscisic acid responsiveness |
| EVM0009496.1 | 1862 | 1908 | ABRE | CACGTG | cis-acting element involved in the abscisic acid responsiveness |
| EVM0009496.1 | 1863 | 1908 | ABRE | ACGTG | cis-acting element involved in the abscisic acid responsiveness |
| EVM0009496.1 | 282 | 327 | TGACG-motif | TGACG | cis-acting regulatory element involved in the MeJA-responsiveness |
| EVM0009496.1 | 1393 | 1438 | TGACG-motif | TGACG | cis-acting regulatory element involved in the MeJA-responsiveness |
| EVM0009496.1 | 679 | 725 | ARE | AAACCA | cis-acting regulatory element essential for the anaerobic induction |
| EVM0009496.1 | 233 | 280 | GATA-motif | GATAGGA | part of a light responsive element |
| EVM0009496.1 | 1624 | 1670 | TCT-motif | TCTTAC | part of a light responsive element |
| EVM0009496.1 | 1958 | 2004 | TCT-motif | TCTTAC | part of a light responsive element |
| EVM0009496.1 | 375 | 422 | MRE | AACCTAA | MYB binding site involved in light responsiveness |
| EVM0009496.1 | 1148 | 1195 | MRE | AACCTAA | MYB binding site involved in light responsiveness |
| EVM0009496.1 | 1889 | 1936 | AuxRR-core | GGTCCAT | cis-acting regulatory element involved in auxin responsiveness |
| EVM0010291.1 | 585 | 631 | LTR | CCGAAA | cis-acting element involved in low-temperature responsiveness |
| EVM0010291.1 | 1700 | 1745 | TGACG-motif | TGACG | cis-acting regulatory element involved in the MeJA-responsiveness |
| EVM0010291.1 | 195 | 240 | ABRE | ACGTG | cis-acting element involved in the abscisic acid responsiveness |
| EVM0010291.1 | 1785 | 1830 | ABRE | ACGTG | cis-acting element involved in the abscisic acid responsiveness |
| EVM0010291.1 | 1700 | 1745 | CGTCA-motif | CGTCA | cis-acting regulatory element involved in the MeJA-responsiveness |
| EVM0010291.1 | 1784 | 1833 | Box II | ACACGTTGT | part of a light responsive element |
| EVM0010291.1 | 151 | 197 | TCT-motif | TCTTAC | part of a light responsive element |
| EVM0010291.1 | 1914 | 1963 | ACE | CTAACGTATT | cis-acting element involved in light responsiveness |
| EVM0010291.1 | 264 | 310 | ARE | AAACCA | cis-acting regulatory element essential for the anaerobic induction |
| EVM0010291.1 | 1685 | 1731 | ARE | AAACCA | cis-acting regulatory element essential for the anaerobic induction |
| EVM0010291.1 | 85 | 131 | Box 4 | ATTAAT | part of a conserved DNA module involved in light responsiveness |
| EVM0010291.1 | 367 | 413 | Box 4 | ATTAAT | part of a conserved DNA module involved in light responsiveness |
| EVM0010291.1 | 441 | 487 | Box 4 | ATTAAT | part of a conserved DNA module involved in light responsiveness |
| EVM0010291.1 | 724 | 770 | Box 4 | ATTAAT | part of a conserved DNA module involved in light responsiveness |
| EVM0010291.1 | 1008 | 1054 | Box 4 | ATTAAT | part of a conserved DNA module involved in light responsiveness |
| EVM0010291.1 | 1012 | 1058 | Box 4 | ATTAAT | part of a conserved DNA module involved in light responsiveness |
| EVM0010291.1 | 1034 | 1080 | Box 4 | ATTAAT | part of a conserved DNA module involved in light responsiveness |
| EVM0010291.1 | 1043 | 1089 | Box 4 | ATTAAT | part of a conserved DNA module involved in light responsiveness |
| EVM0010291.1 | 1093 | 1139 | Box 4 | ATTAAT | part of a conserved DNA module involved in light responsiveness |
| EVM0010291.1 | 1195 | 1241 | Box 4 | ATTAAT | part of a conserved DNA module involved in light responsiveness |
| EVM0010291.1 | 1250 | 1296 | Box 4 | ATTAAT | part of a conserved DNA module involved in light responsiveness |
| EVM0010291.1 | 1296 | 1342 | Box 4 | ATTAAT | part of a conserved DNA module involved in light responsiveness |
| EVM0010291.1 | 1785 | 1831 | G-Box | CACGTT | cis-acting regulatory element involved in light responsiveness |
| EVM0010291.1 | 193 | 243 | G-box | TAACACGTAG | cis-acting regulatory element involved in light responsiveness |
| EVM0010291.1 | 194 | 240 | G-box | TACGTG | cis-acting regulatory element involved in light responsiveness |
| EVM0011973.1 | 754 | 801 | TCCC-motif | TCTCCCT | part of a light responsive element |
| EVM0011973.1 | 1479 | 1524 | TGACG-motif | TGACG | cis-acting regulatory element involved in the MeJA-responsiveness |
| EVM0011973.1 | 1821 | 1866 | TGACG-motif | TGACG | cis-acting regulatory element involved in the MeJA-responsiveness |
| EVM0011973.1 | 1479 | 1524 | CGTCA-motif | CGTCA | cis-acting regulatory element involved in the MeJA-responsiveness |
| EVM0011973.1 | 1821 | 1866 | CGTCA-motif | CGTCA | cis-acting regulatory element involved in the MeJA-responsiveness |
| EVM0011973.1 | 1159 | 1206 | ABRE | TACGGTC | cis-acting element involved in the abscisic acid responsiveness |
| EVM0011973.1 | 1196 | 1241 | ABRE | ACGTG | cis-acting element involved in the abscisic acid responsiveness |
| EVM0011973.1 | 1428 | 1475 | ABRE | TACGGTC | cis-acting element involved in the abscisic acid responsiveness |
| EVM0011973.1 | 1816 | 1861 | ABRE | ACGTG | cis-acting element involved in the abscisic acid responsiveness |
| EVM0011973.1 | 1825 | 1871 | GT1-motif | GGTTAA | light responsive element |
| EVM0011973.1 | 405 | 452 | TATC-box | TATCCCA | cis-acting element involved in gibberellin-responsiveness |
| EVM0011973.1 | 966 | 1017 | SARE | TTCGACCATCTT | cis-acting element involved in salicylic acid responsiveness |
| EVM0011973.1 | 27 | 75 | AE-box | AGAAACAA | part of a module for light response |
| EVM0011973.1 | 1156 | 1202 | G-box | CACGAC | cis-acting regulatory element involved in light responsiveness |
| EVM0011973.1 | 1815 | 1861 | G-box | CACGTC | cis-acting regulatory element involved in light responsiveness |
| EVM0011973.1 | 270 | 317 | MRE | AACCTAA | MYB binding site involved in light responsiveness |
| EVM0011973.1 | 1196 | 1242 | G-Box | CACGTT | cis-acting regulatory element involved in light responsiveness |
| EVM0011973.1 | 1322 | 1368 | TCT-motif | TCTTAC | part of a light responsive element |
| EVM0011973.1 | 1148 | 1198 | I-box | atGATAAGGTC | part of a light responsive element |
| EVM0011973.1 | 661 | 708 | P-box | CCTTTTG | gibberellin-responsive element |
| EVM0011973.1 | 142 | 188 | ARE | AAACCA | cis-acting regulatory element essential for the anaerobic induction |
| EVM0011973.1 | 1502 | 1548 | ARE | AAACCA | cis-acting regulatory element essential for the anaerobic induction |
| EVM0011973.1 | 1124 | 1171 | GATA-motif | GATAGGA | part of a light responsive element |
| EVM0012214.1 | 611 | 659 | chs-CMA2a | TCACTTGA | part of a light responsive element |
| EVM0012214.1 | 1901 | 1950 | TC-rich repeats | GTTTTCTTAC | cis-acting element involved in defense and stress responsiveness |
| EVM0012214.1 | 654 | 700 | ABRE | CACGTG | cis-acting element involved in the abscisic acid responsiveness |
| EVM0012214.1 | 655 | 700 | ABRE | ACGTG | cis-acting element involved in the abscisic acid responsiveness |
| EVM0012214.1 | 1464 | 1509 | ABRE | ACGTG | cis-acting element involved in the abscisic acid responsiveness |
| EVM0012214.1 | 1872 | 1917 | CGTCA-motif | CGTCA | cis-acting regulatory element involved in the MeJA-responsiveness |
| EVM0012214.1 | 1872 | 1917 | TGACG-motif | TGACG | cis-acting regulatory element involved in the MeJA-responsiveness |
| EVM0012214.1 | 654 | 700 | G-box | CACGTG | cis-acting regulatory element involved in light responsiveness |
| EVM0012214.1 | 1464 | 1510 | G-box | TACGTG | cis-acting regulatory element involved in light responsiveness |
| EVM0012214.1 | 55 | 103 | AE-box | AGAAACAA | part of a module for light response |
| EVM0012214.1 | 654 | 700 | G-Box | CACGTG | cis-acting regulatory element involved in light responsiveness |
| EVM0012214.1 | 820 | 868 | chs-CMA1a | TTACTTAA | part of a light responsive element |
| EVM0012214.1 | 135 | 181 | Box 4 | ATTAAT | part of a conserved DNA module involved in light responsiveness |
| EVM0012214.1 | 168 | 214 | Box 4 | ATTAAT | part of a conserved DNA module involved in light responsiveness |
| EVM0012214.1 | 716 | 762 | Box 4 | ATTAAT | part of a conserved DNA module involved in light responsiveness |
| EVM0012214.1 | 1283 | 1329 | Box 4 | ATTAAT | part of a conserved DNA module involved in light responsiveness |
| EVM0012214.1 | 1804 | 1850 | Box 4 | ATTAAT | part of a conserved DNA module involved in light responsiveness |
| EVM0012214.1 | 1938 | 1988 | CAG-motif | GAAAGGCAGAC | part of a light response element |
| EVM0012214.1 | 1652 | 1701 | ACE | CTAACGTATT | cis-acting element involved in light responsiveness |
| EVM0012214.1 | 1334 | 1381 | P-box | CCTTTTG | gibberellin-responsive element |
| EVM0019442.1 | 50 | 96 | MBS | CAACTG | MYB binding site involved in drought-inducibility |
| EVM0019442.1 | 1160 | 1206 | MBS | CAACTG | MYB binding site involved in drought-inducibility |
| EVM0019442.1 | 935 | 981 | LTR | CCGAAA | cis-acting element involved in low-temperature responsiveness |
| EVM0019442.1 | 1469 | 1518 | ABRE | GCAACGTGTC | cis-acting element involved in the abscisic acid responsiveness |
| EVM0019442.1 | 743 | 788 | CGTCA-motif | CGTCA | cis-acting regulatory element involved in the MeJA-responsiveness |
| EVM0019442.1 | 1337 | 1382 | CGTCA-motif | CGTCA | cis-acting regulatory element involved in the MeJA-responsiveness |
| EVM0019442.1 | 1419 | 1464 | CGTCA-motif | CGTCA | cis-acting regulatory element involved in the MeJA-responsiveness |
| EVM0019442.1 | 743 | 788 | TGACG-motif | TGACG | cis-acting regulatory element involved in the MeJA-responsiveness |
| EVM0019442.1 | 1337 | 1382 | TGACG-motif | TGACG | cis-acting regulatory element involved in the MeJA-responsiveness |
| EVM0019442.1 | 1419 | 1464 | TGACG-motif | TGACG | cis-acting regulatory element involved in the MeJA-responsiveness |
| EVM0019442.1 | 1714 | 1760 | Box 4 | ATTAAT | part of a conserved DNA module involved in light responsiveness |
| EVM0019442.1 | 1937 | 1983 | Box 4 | ATTAAT | part of a conserved DNA module involved in light responsiveness |
| EVM0019442.1 | 1292 | 1342 | GATA-motif | AAGATAAGATT | part of a light responsive element |
| EVM0019442.1 | 1063 | 1109 | ARE | AAACCA | cis-acting regulatory element essential for the anaerobic induction |
| EVM0019442.1 | 1623 | 1669 | ARE | AAACCA | cis-acting regulatory element essential for the anaerobic induction |
| EVM0019442.1 | 898 | 944 | G-box | CACGAC | cis-acting regulatory element involved in light responsiveness |
| EVM0019814.1 | 816 | 864 | LAMP-element | CTTTATCA | part of a light responsive element |
| EVM0019814.1 | 52 | 98 | LTR | CCGAAA | cis-acting element involved in low-temperature responsiveness |
| EVM0019814.1 | 115 | 164.5 | Gap-box | CAAATGAA(A/G)A | part of a light responsive element |
| EVM0019814.1 | 1508 | 1557 | TCA-element | CCATCTTTTT | cis-acting element involved in salicylic acid responsiveness |
| EVM0019814.1 | 1248 | 1293 | TGACG-motif | TGACG | cis-acting regulatory element involved in the MeJA-responsiveness |
| EVM0019814.1 | 1685 | 1730 | TGACG-motif | TGACG | cis-acting regulatory element involved in the MeJA-responsiveness |
| EVM0019814.1 | 1217 | 1263 | TGA-element | AACGAC | auxin-responsive element |
| EVM0019814.1 | 1613 | 1659 | TGA-element | AACGAC | auxin-responsive element |
| EVM0019814.1 | 1499 | 1547 | GA-motif | ATAGATAA | part of a light responsive element |
| EVM0019814.1 | 1627 | 1673 | GT1-motif | GGTTAA | light responsive element |
| EVM0019814.1 | 1248 | 1293 | CGTCA-motif | CGTCA | cis-acting regulatory element involved in the MeJA-responsiveness |
| EVM0019814.1 | 1685 | 1730 | CGTCA-motif | CGTCA | cis-acting regulatory element involved in the MeJA-responsiveness |
| EVM0019814.1 | 789 | 838 | ABRE | GACACGTGGC | cis-acting element involved in the abscisic acid responsiveness |
| EVM0019814.1 | 791 | 837 | ABRE | CACGTG | cis-acting element involved in the abscisic acid responsiveness |
| EVM0019814.1 | 792 | 837 | ABRE | ACGTG | cis-acting element involved in the abscisic acid responsiveness |
| EVM0019814.1 | 866 | 912 | ABRE | CACGTG | cis-acting element involved in the abscisic acid responsiveness |
| EVM0019814.1 | 867 | 912 | ABRE | ACGTG | cis-acting element involved in the abscisic acid responsiveness |
| EVM0019814.1 | 1387 | 1436 | ABRE | GACACGTGGC | cis-acting element involved in the abscisic acid responsiveness |
| EVM0019814.1 | 1389 | 1435 | ABRE | CACGTG | cis-acting element involved in the abscisic acid responsiveness |
| EVM0019814.1 | 1390 | 1435 | ABRE | ACGTG | cis-acting element involved in the abscisic acid responsiveness |
| EVM0019814.1 | 1619 | 1669 | 3-AF1 binding site | TAAGAGAGGAA | light responsive element |
| EVM0019814.1 | 1431 | 1477 | Box 4 | ATTAAT | part of a conserved DNA module involved in light responsiveness |
| EVM0019814.1 | 9 | 58 | ATCT-motif | AATCTAATCC | part of a conserved DNA module involved in light responsiveness |
| EVM0019814.1 | 1331 | 1380 | GATA-motif | AAGGATAAGG | part of a light responsive element |
| EVM0019814.1 | 1081 | 1127 | ARE | AAACCA | cis-acting regulatory element essential for the anaerobic induction |
| EVM0019814.1 | 1405 | 1455 | L-box | ATCCCACCTAC | part of a light responsive element |
| EVM0019814.1 | 1548 | 1595 | MRE | AACCTAA | MYB binding site involved in light responsiveness |
| EVM0019814.1 | 668 | 714 | G-box | CACGAC | cis-acting regulatory element involved in light responsiveness |
| EVM0019814.1 | 790 | 838 | G-box | ACACGTGT | cis-acting regulatory element involved in light responsiveness |
| EVM0019814.1 | 791 | 837 | G-box | CACGTG | cis-acting regulatory element involved in light responsiveness |
| EVM0019814.1 | 865 | 913 | G-box | ACACGTGT | cis-acting regulatory element involved in light responsiveness |
| EVM0019814.1 | 866 | 912 | G-box | CACGTG | cis-acting regulatory element involved in light responsiveness |
| EVM0019814.1 | 1389 | 1435 | G-box | CACGTG | cis-acting regulatory element involved in light responsiveness |
| EVM0019814.1 | 791 | 837 | G-Box | CACGTG | cis-acting regulatory element involved in light responsiveness |
| EVM0019814.1 | 866 | 912 | G-Box | CACGTG | cis-acting regulatory element involved in light responsiveness |
| EVM0019814.1 | 1389 | 1435 | G-Box | CACGTG | cis-acting regulatory element involved in light responsiveness |
| EVM0023331.1 | 578 | 626 | chs-CMA2a | TCACTTGA | part of a light responsive element |
| EVM0023331.1 | 1451 | 1497 | ABRE | CACGTG | cis-acting element involved in the abscisic acid responsiveness |
| EVM0023331.1 | 1452 | 1497 | ABRE | ACGTG | cis-acting element involved in the abscisic acid responsiveness |
| EVM0023331.1 | 1469 | 1515 | ABRE | CACGTG | cis-acting element involved in the abscisic acid responsiveness |
| EVM0023331.1 | 1470 | 1515 | ABRE | ACGTG | cis-acting element involved in the abscisic acid responsiveness |
| EVM0023331.1 | 1581 | 1626 | ABRE | ACGTG | cis-acting element involved in the abscisic acid responsiveness |
| EVM0023331.1 | 1222 | 1267 | CGTCA-motif | CGTCA | cis-acting regulatory element involved in the MeJA-responsiveness |
| EVM0023331.1 | 1563 | 1608 | CGTCA-motif | CGTCA | cis-acting regulatory element involved in the MeJA-responsiveness |
| EVM0023331.1 | 145 | 191 | GT1-motif | GGTTAA | light responsive element |
| EVM0023331.1 | 1298 | 1344 | GT1-motif | GGTTAA | light responsive element |
| EVM0023331.1 | 1222 | 1267 | TGACG-motif | TGACG | cis-acting regulatory element involved in the MeJA-responsiveness |
| EVM0023331.1 | 1563 | 1608 | TGACG-motif | TGACG | cis-acting regulatory element involved in the MeJA-responsiveness |
| EVM0023331.1 | 962 | 1008 | MBS | CAACTG | MYB binding site involved in drought-inducibility |
| EVM0023331.1 | 998 | 1044 | MBS | CAACTG | MYB binding site involved in drought-inducibility |
| EVM0023331.1 | 1640 | 1686 | LTR | CCGAAA | cis-acting element involved in low-temperature responsiveness |
| EVM0023331.1 | 1449 | 1498 | G-box | GCCACGTGGA | cis-acting regulatory element involved in light responsiveness |
| EVM0023331.1 | 1451 | 1497 | G-box | CACGTG | cis-acting regulatory element involved in light responsiveness |
| EVM0023331.1 | 1469 | 1515 | G-box | CACGTG | cis-acting regulatory element involved in light responsiveness |
| EVM0023331.1 | 1581 | 1627 | G-box | CACGTC | cis-acting regulatory element involved in light responsiveness |
| EVM0023331.1 | 1451 | 1497 | G-Box | CACGTG | cis-acting regulatory element involved in light responsiveness |
| EVM0023331.1 | 1469 | 1515 | G-Box | CACGTG | cis-acting regulatory element involved in light responsiveness |
| EVM0023331.1 | 290 | 336 | Box 4 | ATTAAT | part of a conserved DNA module involved in light responsiveness |
| EVM0023331.1 | 788 | 834 | Box 4 | ATTAAT | part of a conserved DNA module involved in light responsiveness |
| EVM0023331.1 | 818 | 864 | Box 4 | ATTAAT | part of a conserved DNA module involved in light responsiveness |
| EVM0023331.1 | 1780 | 1826 | Box 4 | ATTAAT | part of a conserved DNA module involved in light responsiveness |
| EVM0023331.1 | 1602 | 1650 | I-box | AGATAAGG | part of a light responsive element |
| EVM0023331.1 | 1459 | 1506 | GATA-motif | GATAGGG | part of a light responsive element |
| EVM0023331.1 | 1239 | 1285 | TCT-motif | TCTTAC | part of a light responsive element |
| EVM0024063.1 | 743 | 789 | TGA-element | AACGAC | auxin-responsive element |
| EVM0024063.1 | 245 | 290 | TGACG-motif | TGACG | cis-acting regulatory element involved in the MeJA-responsiveness |
| EVM0024063.1 | 1658 | 1706 | GA-motif | ATAGATAA | part of a light responsive element |
| EVM0024063.1 | 245 | 290 | CGTCA-motif | CGTCA | cis-acting regulatory element involved in the MeJA-responsiveness |
| EVM0024063.1 | 1355 | 1404 | TCA-element | CCATCTTTTT | cis-acting element involved in salicylic acid responsiveness |
| EVM0024063.1 | 736 | 785 | TC-rich repeats | GTTTTCTTAC | cis-acting element involved in defense and stress responsiveness |
| EVM0024063.1 | 827 | 873 | Sp1 | GGGCGG | light responsive element |
| EVM0024063.1 | 979 | 1026 | MRE | AACCTAA | MYB binding site involved in light responsiveness |
| EVM0024063.1 | 132 | 178 | G-box | CACGAC | cis-acting regulatory element involved in light responsiveness |
| EVM0024063.1 | 875 | 921 | TCT-motif | TCTTAC | part of a light responsive element |
| EVM0024063.1 | 765 | 811 | ARE | AAACCA | cis-acting regulatory element essential for the anaerobic induction |
| EVM0024063.1 | 511 | 557 | Box 4 | ATTAAT | part of a conserved DNA module involved in light responsiveness |
| EVM0024063.1 | 955 | 1001 | Box 4 | ATTAAT | part of a conserved DNA module involved in light responsiveness |
| EVM0024063.1 | 1604 | 1650 | Box 4 | ATTAAT | part of a conserved DNA module involved in light responsiveness |
| EVM0024063.1 | 1940 | 1986 | Box 4 | ATTAAT | part of a conserved DNA module involved in light responsiveness |
| EVM0026412.2 | 1635 | 1684 | TC-rich repeats | ATTCTCTAAC | cis-acting element involved in defense and stress responsiveness |
| EVM0026412.2 | 861 | 907 | TGA-element | AACGAC | auxin-responsive element |
| EVM0026412.2 | 551 | 596 | TGACG-motif | TGACG | cis-acting regulatory element involved in the MeJA-responsiveness |
| EVM0026412.2 | 123 | 169 | GT1-motif | GGTTAA | light responsive element |
| EVM0026412.2 | 535 | 581 | ABRE | CACGTG | cis-acting element involved in the abscisic acid responsiveness |
| EVM0026412.2 | 536 | 581 | ABRE | ACGTG | cis-acting element involved in the abscisic acid responsiveness |
| EVM0026412.2 | 553 | 598 | ABRE | ACGTG | cis-acting element involved in the abscisic acid responsiveness |
| EVM0026412.2 | 1403 | 1448 | ABRE | ACGTG | cis-acting element involved in the abscisic acid responsiveness |
| EVM0026412.2 | 1447 | 1492 | ABRE | ACGTG | cis-acting element involved in the abscisic acid responsiveness |
| EVM0026412.2 | 1487 | 1532 | ABRE | ACGTG | cis-acting element involved in the abscisic acid responsiveness |
| EVM0026412.2 | 551 | 596 | CGTCA-motif | CGTCA | cis-acting regulatory element involved in the MeJA-responsiveness |
| EVM0026412.2 | 153 | 199 | Box 4 | ATTAAT | part of a conserved DNA module involved in light responsiveness |
| EVM0026412.2 | 283 | 329 | Box 4 | ATTAAT | part of a conserved DNA module involved in light responsiveness |
| EVM0026412.2 | 649 | 695 | Box 4 | ATTAAT | part of a conserved DNA module involved in light responsiveness |
| EVM0026412.2 | 820 | 866 | Box 4 | ATTAAT | part of a conserved DNA module involved in light responsiveness |
| EVM0026412.2 | 1718 | 1764 | Box 4 | ATTAAT | part of a conserved DNA module involved in light responsiveness |
| EVM0026412.2 | 1111 | 1157 | TCT-motif | TCTTAC | part of a light responsive element |
| EVM0026412.2 | 1142 | 1188 | TCT-motif | TCTTAC | part of a light responsive element |
| EVM0026412.2 | 116 | 162 | ARE | AAACCA | cis-acting regulatory element essential for the anaerobic induction |
| EVM0026412.2 | 134 | 180 | ARE | AAACCA | cis-acting regulatory element essential for the anaerobic induction |
| EVM0026412.2 | 199 | 245 | ARE | AAACCA | cis-acting regulatory element essential for the anaerobic induction |
| EVM0026412.2 | 534 | 582 | G-box | ACACGTGT | cis-acting regulatory element involved in light responsiveness |
| EVM0026412.2 | 535 | 581 | G-box | CACGTG | cis-acting regulatory element involved in light responsiveness |
| EVM0026412.2 | 552 | 598 | G-box | CACGTC | cis-acting regulatory element involved in light responsiveness |
| EVM0026412.2 | 1446 | 1492 | G-box | CACGTC | cis-acting regulatory element involved in light responsiveness |
| EVM0026412.2 | 535 | 581 | G-Box | CACGTG | cis-acting regulatory element involved in light responsiveness |
| EVM0026412.2 | 1403 | 1449 | G-Box | CACGTT | cis-acting regulatory element involved in light responsiveness |
| EVM0026412.2 | 1487 | 1533 | G-Box | CACGTT | cis-acting regulatory element involved in light responsiveness |
| EVM0028473.1 | 1815 | 1861 | LTR | CCGAAA | cis-acting element involved in low-temperature responsiveness |
| EVM0028473.1 | 1377 | 1423 | GT1-motif | GGTTAA | light responsive element |
| EVM0028473.1 | 1143 | 1191 | GA-motif | ATAGATAA | part of a light responsive element |
| EVM0028473.1 | 358 | 404 | ABRE | CACGTG | cis-acting element involved in the abscisic acid responsiveness |
| EVM0028473.1 | 359 | 404 | ABRE | ACGTG | cis-acting element involved in the abscisic acid responsiveness |
| EVM0028473.1 | 1746 | 1792 | ABRE | CACGTG | cis-acting element involved in the abscisic acid responsiveness |
| EVM0028473.1 | 1747 | 1792 | ABRE | ACGTG | cis-acting element involved in the abscisic acid responsiveness |
| EVM0028473.1 | 1065 | 1110 | CGTCA-motif | CGTCA | cis-acting regulatory element involved in the MeJA-responsiveness |
| EVM0028473.1 | 1065 | 1110 | TGACG-motif | TGACG | cis-acting regulatory element involved in the MeJA-responsiveness |
| EVM0028473.1 | 914 | 963 | TCA-element | CCATCTTTTT | cis-acting element involved in salicylic acid responsiveness |
| EVM0028473.1 | 57 | 104 | GATA-motif | GATAGGA | part of a light responsive element |
| EVM0028473.1 | 107 | 156 | ACE | CTAACGTATT | cis-acting element involved in light responsiveness |
| EVM0028473.1 | 671 | 720 | ACE | CTAACGTATT | cis-acting element involved in light responsiveness |
| EVM0028473.1 | 394 | 440 | ARE | AAACCA | cis-acting regulatory element essential for the anaerobic induction |
| EVM0028473.1 | 753 | 799 | ARE | AAACCA | cis-acting regulatory element essential for the anaerobic induction |
| EVM0028473.1 | 911 | 957 | ARE | AAACCA | cis-acting regulatory element essential for the anaerobic induction |
| EVM0028473.1 | 998 | 1044 | ARE | AAACCA | cis-acting regulatory element essential for the anaerobic induction |
| EVM0028473.1 | 1160 | 1206 | ARE | AAACCA | cis-acting regulatory element essential for the anaerobic induction |
| EVM0028473.1 | 1915 | 1961 | ARE | AAACCA | cis-acting regulatory element essential for the anaerobic induction |
| EVM0028473.1 | 1599 | 1646 | P-box | CCTTTTG | gibberellin-responsive element |
| EVM0028473.1 | 732 | 778 | TCT-motif | TCTTAC | part of a light responsive element |
| EVM0028473.1 | 1828 | 1874 | TCT-motif | TCTTAC | part of a light responsive element |
| EVM0028473.1 | 119 | 165 | Box 4 | ATTAAT | part of a conserved DNA module involved in light responsiveness |
| EVM0028473.1 | 358 | 404 | G-Box | CACGTG | cis-acting regulatory element involved in light responsiveness |
| EVM0028473.1 | 1746 | 1792 | G-Box | CACGTG | cis-acting regulatory element involved in light responsiveness |
| EVM0028473.1 | 1473 | 1520 | AuxRR-core | GGTCCAT | cis-acting regulatory element involved in auxin responsiveness |
| EVM0028473.1 | 358 | 404 | G-box | CACGTG | cis-acting regulatory element involved in light responsiveness |
| EVM0028473.1 | 1746 | 1792 | G-box | CACGTG | cis-acting regulatory element involved in light responsiveness |
| EVM0028473.1 | 1218 | 1266 | AE-box | AGAAACAA | part of a module for light response |
| EVM0029236.1 | 1321 | 1367 | Box 4 | ATTAAT | part of a conserved DNA module involved in light responsiveness |
| EVM0029236.1 | 1704 | 1750 | Box 4 | ATTAAT | part of a conserved DNA module involved in light responsiveness |
| EVM0029236.1 | 1197 | 1244 | GARE-motif | TCTGTTG | gibberellin-responsive element |
| EVM0029236.1 | 62 | 108 | ARE | AAACCA | cis-acting regulatory element essential for the anaerobic induction |
| EVM0029236.1 | 1971 | 2018 | P-box | CCTTTTG | gibberellin-responsive element |
| EVM0029236.1 | 1213 | 1262 | ATC-motif | TGCTATCCA | part of a conserved DNA module involved in light responsiveness |
| EVM0029236.1 | 1566 | 1612 | G-box | CACGTG | cis-acting regulatory element involved in light responsiveness |
| EVM0029236.1 | 1664 | 1710 | G-box | CACGTG | cis-acting regulatory element involved in light responsiveness |
| EVM0029236.1 | 1849 | 1895 | G-box | TACGTG | cis-acting regulatory element involved in light responsiveness |
| EVM0029236.1 | 1566 | 1612 | G-Box | CACGTG | cis-acting regulatory element involved in light responsiveness |
| EVM0029236.1 | 1664 | 1710 | G-Box | CACGTG | cis-acting regulatory element involved in light responsiveness |
| EVM0029236.1 | 754 | 800 | Sp1 | GGGCGG | light responsive element |
| EVM0029236.1 | 1153 | 1202 | TCA-element | CCATCTTTTT | cis-acting element involved in salicylic acid responsiveness |
| EVM0029236.1 | 1429 | 1478 | TCA-element | CCATCTTTTT | cis-acting element involved in salicylic acid responsiveness |
| EVM0029236.1 | 155 | 201 | TGA-element | AACGAC | auxin-responsive element |
| EVM0029236.1 | 794 | 840 | TGA-element | AACGAC | auxin-responsive element |
| EVM0029236.1 | 1566 | 1612 | ABRE | CACGTG | cis-acting element involved in the abscisic acid responsiveness |
| EVM0029236.1 | 1567 | 1612 | ABRE | ACGTG | cis-acting element involved in the abscisic acid responsiveness |
| EVM0029236.1 | 1664 | 1710 | ABRE | CACGTG | cis-acting element involved in the abscisic acid responsiveness |
| EVM0029236.1 | 1665 | 1710 | ABRE | ACGTG | cis-acting element involved in the abscisic acid responsiveness |
| EVM0029236.1 | 1849 | 1894 | ABRE | ACGTG | cis-acting element involved in the abscisic acid responsiveness |
| EVM0029236.1 | 447 | 495 | GA-motif | ATAGATAA | part of a light responsive element |
| EVM0030977.1 | 45 | 91 | G-box | CACGTC | cis-acting regulatory element involved in light responsiveness |
| EVM0030977.1 | 49 | 98 | G-box | GCCACGTGGA | cis-acting regulatory element involved in light responsiveness |
| EVM0030977.1 | 1255 | 1301 | Box 4 | ATTAAT | part of a conserved DNA module involved in light responsiveness |
| EVM0030977.1 | 1640 | 1686 | ARE | AAACCA | cis-acting regulatory element essential for the anaerobic induction |
| EVM0030977.1 | 1926 | 1973 | P-box | CCTTTTG | gibberellin-responsive element |
| EVM0030977.1 | 1606 | 1655 | TC-rich repeats | GTTTTCTTAC | cis-acting element involved in defense and stress responsiveness |
| EVM0030977.1 | 1912 | 1961 | TC-rich repeats | ATTCTCTAAC | cis-acting element involved in defense and stress responsiveness |
| EVM0030977.1 | 45 | 90 | ABRE | ACGTG | cis-acting element involved in the abscisic acid responsiveness |
| EVM0030977.1 | 62 | 111 | ABRE | GCCGCGTGGC | cis-acting element involved in the abscisic acid responsiveness |
| EVM0030977.1 | 830 | 876 | GT1-motif | GGTTAA | light responsive element |
| EVM0030977.1 | 1917 | 1963 | GT1-motif | GGTTAA | light responsive element |
| EVM0030977.1 | 1774 | 1820 | TGA-element | AACGAC | auxin-responsive element |
| EVM0030977.1 | 120 | 166 | MBS | CAACTG | MYB binding site involved in drought-inducibility |
| EVM0032786.1 | 1086 | 1131 | TGACG-motif | TGACG | cis-acting regulatory element involved in the MeJA-responsiveness |
| EVM0032786.1 | 1531 | 1576 | TGACG-motif | TGACG | cis-acting regulatory element involved in the MeJA-responsiveness |
| EVM0032786.1 | 1086 | 1131 | CGTCA-motif | CGTCA | cis-acting regulatory element involved in the MeJA-responsiveness |
| EVM0032786.1 | 1531 | 1576 | CGTCA-motif | CGTCA | cis-acting regulatory element involved in the MeJA-responsiveness |
| EVM0032786.1 | 104 | 149 | ABRE | ACGTG | cis-acting element involved in the abscisic acid responsiveness |
| EVM0032786.1 | 648 | 694 | ABRE | CACGTG | cis-acting element involved in the abscisic acid responsiveness |
| EVM0032786.1 | 649 | 694 | ABRE | ACGTG | cis-acting element involved in the abscisic acid responsiveness |
| EVM0032786.1 | 1174 | 1219 | ABRE | ACGTG | cis-acting element involved in the abscisic acid responsiveness |
| EVM0032786.1 | 1765 | 1810 | ABRE | ACGTG | cis-acting element involved in the abscisic acid responsiveness |
| EVM0032786.1 | 1405 | 1451 | LTR | CCGAAA | cis-acting element involved in low-temperature responsiveness |
| EVM0032786.1 | 502 | 548 | G-box | CACGAC | cis-acting regulatory element involved in light responsiveness |
| EVM0032786.1 | 647 | 695 | G-box | ACACGTGT | cis-acting regulatory element involved in light responsiveness |
| EVM0032786.1 | 648 | 694 | G-box | CACGTG | cis-acting regulatory element involved in light responsiveness |
| EVM0032786.1 | 1173 | 1219 | G-box | TACGTG | cis-acting regulatory element involved in light responsiveness |
| EVM0032786.1 | 1433 | 1480 | AuxRR-core | GGTCCAT | cis-acting regulatory element involved in auxin responsiveness |
| EVM0032786.1 | 103 | 149 | G-Box | CACGTT | cis-acting regulatory element involved in light responsiveness |
| EVM0032786.1 | 648 | 694 | G-Box | CACGTG | cis-acting regulatory element involved in light responsiveness |
| EVM0032786.1 | 1765 | 1811 | G-Box | CACGTT | cis-acting regulatory element involved in light responsiveness |
| EVM0032786.1 | 418 | 464 | Box 4 | ATTAAT | part of a conserved DNA module involved in light responsiveness |
| EVM0032786.1 | 768 | 814 | Box 4 | ATTAAT | part of a conserved DNA module involved in light responsiveness |
| EVM0032786.1 | 1475 | 1521 | Box 4 | ATTAAT | part of a conserved DNA module involved in light responsiveness |
| EVM0032786.1 | 893 | 941 | chs-CMA1a | TTACTTAA | part of a light responsive element |
| EVM0032786.1 | 421 | 470 | ATCT-motif | AATCTAATCC | part of a conserved DNA module involved in light responsiveness |
| EVM0032786.1 | 510 | 556 | TCT-motif | TCTTAC | part of a light responsive element |
| EVM0032786.1 | 1971 | 2017 | ARE | AAACCA | cis-acting regulatory element essential for the anaerobic induction |
| EVM0032786.1 | 117 | 164 | P-box | CCTTTTG | gibberellin-responsive element |

| **Table S5** **\|** The expression level of CCD genes in fruits, leaves, stems and flowers of F. suspensa. | | | | | | | | | | | | | |
| --- | --- | --- | --- | --- | --- | --- | --- | --- | --- | --- | --- | --- | --- |
| Gene | Gene ID | Fruit-1 | Fruit-2 | Fruit-3 | Stem-1 | Stem-2 | Stem-3 | Leaf-1 | Leaf-2 | Leaf-3 | Flower-1 | Flower-2 | Flower-3 |
| *FsCCD1-1* | EVM0026412.2 | 51.132 | 35.126 | 55.348 | 13.370 | 24.732 | 31.656 | 45.948 | 38.210 | 82.768 | 27.381 | 29.700 | 31.104 |
| *FsCCD1-2* | EVM0032786.1 | 7.555 | 7.209 | 9.279 | 1.712 | 2.387 | 2.659 | 4.416 | 3.606 | 6.990 | 3.521 | 5.743 | 4.612 |
| *FsCCD1-3* | EVM0011973.1 | 84.067 | 43.971 | 48.965 | 42.028 | 44.911 | 57.685 | 91.058 | 63.345 | 86.814 | 53.612 | 53.791 | 57.149 |
| *FsCCD1-4* | EVM0019814.1 | 0.000 | 0.833 | 0.368 | 0.000 | 0.096 | 1.111 | 0.062 | 0.077 | 0.505 | 0.229 | 0.428 | 0.474 |
| *FsCCD4-1* | EVM0012214.1 | 0.278 | 0.125 | 0.129 | 0.368 | 0.134 | 0.418 | 0.000 | 0.000 | 0.000 | 28.492 | 30.152 | 29.886 |
| *FsCCD4-2* | EVM0029236.1 | 0.000 | 0.147 | 0.000 | 0.000 | 0.000 | 0.000 | 0.000 | 0.095 | 0.000 | 3.224 | 4.129 | 2.576 |
| *FsCCD4-3* | EVM0019442.1 | 2.308 | 3.046 | 2.417 | 1.926 | 0.692 | 5.074 | 5.183 | 9.448 | 11.244 | 2.609 | 3.577 | 3.497 |
| *FsCCD4-4* | EVM0030977.1 | 1.360 | 1.443 | 0.989 | 0.353 | 0.232 | 1.755 | 2.047 | 3.497 | 5.027 | 2.609 | 3.577 | 3.497 |
| *FsCCD4-5* | EVM0024063.1 | 0.397 | 0.036 | 0.061 | 0.201 | 0.000 | 0.000 | 0.183 | 0.000 | 0.000 | 0.286 | 0.000 | 0.000 |
| *FsCCD7* | EVM0010291.1 | 0.000 | 0.353 | 0.206 | 0.000 | 0.018 | 0.036 | 0.000 | 0.000 | 0.000 | 5.517 | 3.148 | 3.465 |
| *FsCCD8* | EVM0008236.1 | 0.032 | 0.000 | 0.000 | 0.000 | 0.017 | 1.042 | 0.077 | 0.977 | 3.426 | 0.018 | 0.000 | 0.000 |
| *FsNCED1-1* | EVM0003923.1 | 10.238 | 14.889 | 4.077 | 94.787 | 57.118 | 10.054 | 12.754 | 18.174 | 48.961 | 2.990 | 2.898 | 3.235 |
| *FsNCED1-2* | EVM0009496.1 | 2.375 | 1.332 | 0.900 | 29.345 | 6.973 | 1.500 | 4.426 | 3.611 | 5.795 | 1.452 | 1.434 | 1.125 |
| *FsNCED5-1* | EVM0028473.1 | 1.675 | 0.476 | 4.406 | 5.512 | 4.150 | 1.551 | 0.556 | 0.444 | 1.276 | 0.000 | 0.000 | 0.000 |
| *FsNCED5-2* | EVM0005619.1 | 0.765 | 1.762 | 1.323 | 1.781 | 6.894 | 2.163 | 0.064 | 0.104 | 0.000 | 0.484 | 0.511 | 0.447 |
| *FsNCED6* | EVM0023331.1 | 0.714 | 3.191 | 3.833 | 0.327 | 0.000 | 0.000 | 0.000 | 0.000 | 0.000 | 0.000 | 0.028 | 0.032 |

| **Table S6** **\| The expression level of *CCD* genes in leaves of *F. suspensa* in response to cold stress.** | | | | | | | |
| --- | --- | --- | --- | --- | --- | --- | --- |
| Gene | Gene ID | Control-1 | Control-2 | Control-3 | Cold treatment-1 | Cold treatment-2 | Cold treatment-3 |
| *FsCCD1-1* | EVM0026412.2 | 150.269 | 139.403 | 166.005 | 155.484 | 122.200 | 122.702 |
| *FsCCD1-2* | EVM0032786.1 | 5.219 | 0.406 | 0.577 | 5.210 | 4.120 | 4.870 |
| *FsCCD1-3* | EVM0011973.1 | 138.411 | 148.580 | 147.106 | 139.653 | 95.070 | 71.899 |
| *FsCCD1-4* | EVM0019814.1 | 0.938 | 2.510 | 2.145 | 2.453 | 1.596 | 0.299 |
| *FsCCD4-1* | EVM0012214.1 | 0.000 | 0.000 | 0.000 | 0.000 | 0.163 | 0.000 |
| *FsCCD4-2* | EVM0029236.1 | 0.000 | 0.000 | 0.000 | 0.000 | 0.000 | 0.000 |
| *FsCCD4-3* | EVM0019442.1 | 497.445 | 372.706 | 405.256 | 315.763 | 226.942 | 302.452 |
| *FsCCD4-4* | EVM0030977.1 | 391.262 | 266.706 | 280.601 | 250.147 | 158.004 | 188.361 |
| *FsCCD4-5* | EVM0024063.1 | 0.000 | 0.000 | 0.000 | 0.000 | 0.000 | 0.000 |
| *FsCCD7* | EVM0010291.1 | 0.000 | 0.000 | 0.000 | 0.000 | 0.000 | 0.000 |
| *FsCCD8* | EVM0008236.1 | 1.128 | 1.561 | 1.632 | 0.804 | 0.414 | 0.449 |
| *FsNCED1-1* | EVM0003923.1 | 2.305 | 9.578 | 1.658 | 28.092 | 40.257 | 68.082 |
| *FsNCED1-2* | EVM0009496.1 | 1.059 | 2.801 | 0.842 | 24.521 | 18.081 | 32.533 |
| *FsNCED5-1* | EVM0028473.1 | 0.800 | 0.906 | 0.490 | 3.587 | 7.367 | 7.352 |
| *FsNCED5-2* | EVM0005619.1 | 0.000 | 0.000 | 0.000 | 0.420 | 0.289 | 0.498 |
| *FsNCED6* | EVM0023331.1 | 0.000 | 0.000 | 0.000 | 0.000 | 0.000 | 0.000 |

| **Table S7** **\| The expression level of *CCD* genes in leaves of *F. suspensa* in response to drought stress.** | | | | | | | |
| --- | --- | --- | --- | --- | --- | --- | --- |
| Gene | Gene ID | Control-1 | Control-2 | Control-3 | Drought treatment-1 | Drought treatment-2 | Drought treatment-3 |
| *FsCCD1-1* | EVM0026412.2 | 59.624 | 61.664 | 69.960 | 122.399 | 116.414 | 121.421 |
| *FsCCD1-2* | EVM0032786.1 | 3.035 | 1.811 | 2.810 | 3.455 | 6.094 | 4.820 |
| *FsCCD1-3* | EVM0011973.1 | 109.858 | 123.873 | 198.344 | 49.924 | 95.018 | 107.819 |
| *FsCCD1-4* | EVM0019814.1 | 0.296 | 0.313 | 3.037 | 1.493 | 1.320 | 5.030 |
| *FsCCD4-1* | EVM0012214.1 | 0.055 | 0.091 | 0.000 | 0.000 | 0.021 | 0.042 |
| *FsCCD4-2* | EVM0029236.1 | 0.000 | 0.000 | 0.000 | 0.000 | 0.000 | 0.000 |
| *FsCCD4-3* | EVM0019442.1 | 855.743 | 650.861 | 764.542 | 203.015 | 79.889 | 101.847 |
| *FsCCD4-4* | EVM0030977.1 | 892.242 | 541.662 | 732.805 | 96.744 | 82.920 | 109.405 |
| *FsCCD4-5* | EVM0024063.1 | 0.000 | 0.000 | 0.048 | 0.000 | 0.000 | 0.034 |
| *FsCCD7* | EVM0010291.1 | 0.027 | 0.000 | 0.000 | 0.000 | 0.000 | 0.000 |
| *FsCCD8* | EVM0008236.1 | 0.524 | 1.347 | 0.886 | 0.035 | 0.535 | 0.000 |
| *FsNCED1-1* | EVM0003923.1 | 8.753 | 8.529 | 6.940 | 62.459 | 48.175 | 93.818 |
| *FsNCED1-2* | EVM0009496.1 | 3.865 | 1.611 | 1.680 | 7.630 | 5.117 | 2.702 |
| *FsNCED5-1* | EVM0028473.1 | 0.572 | 0.573 | 0.386 | 0.412 | 0.293 | 0.158 |
| *FsNCED5-2* | EVM0005619.1 | 0.192 | 0.000 | 0.089 | 0.000 | 0.000 | 0.164 |
| *FsNCED6* | EVM0023331.1 | 0.000 | 0.000 | 0.000 | 0.000 | 0.000 | 0.021 |

# 2 Supplementary Figures


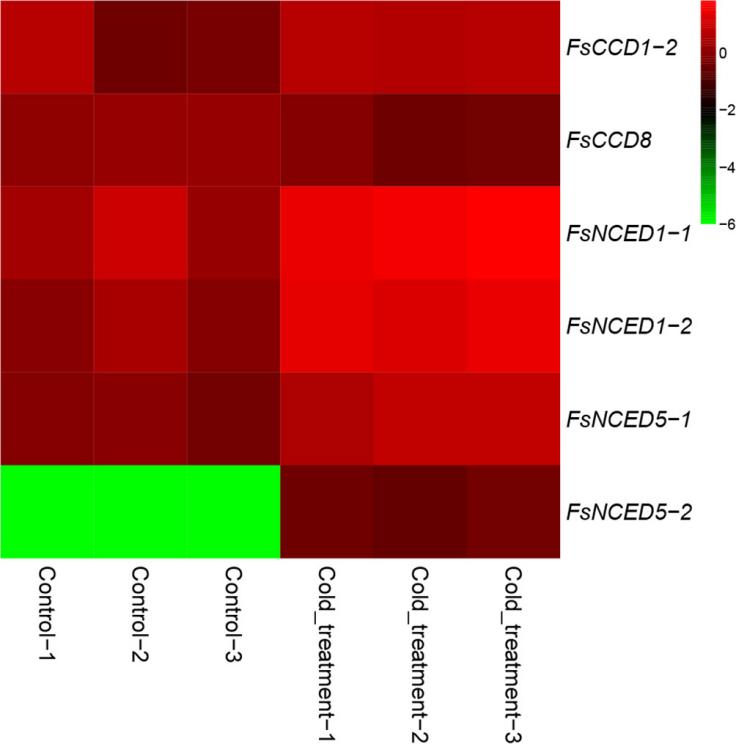


**Fig. S1 Heat map of *CCD* gene expression (Log_10_FPKM) in response to cold stress of** *Forsythia suspensa***. Control-1 to Control-3 and Cold_treatment-1 to Cold_treatment-3 indicate the three biological replicates from the control and cold treatment groups.**

**
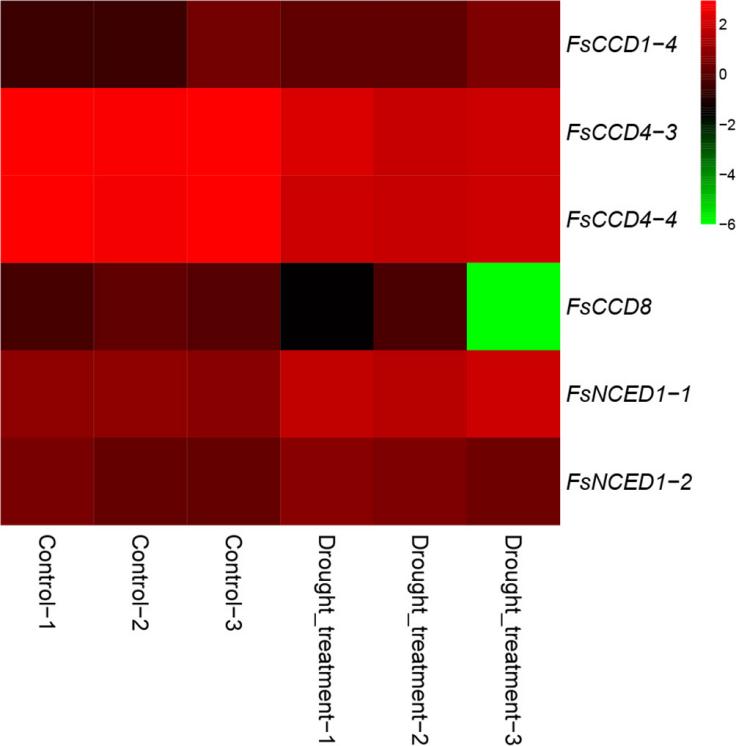
**

**Fig. S2 Heat map of *WRKY* gene expression (Log_10_FPKM) in response to drought stress of** *Forsythia suspensa***. Control-1 to Control-3 and Drought_treatment-1 to Drought_treatment-3 indicate the three biological replicates from the control and drought treatment groups.**
